# Heterotrophy among Cyanobacteria

**DOI:** 10.1021/acsomega.3c02205

**Published:** 2023-09-06

**Authors:** Ronald Stebegg, Georg Schmetterer, Annette Rompel

**Affiliations:** Universität Wien, Fakultät für Chemie, Institut für Biophysikalische Chemie, 1090 Wien, Austria

## Abstract

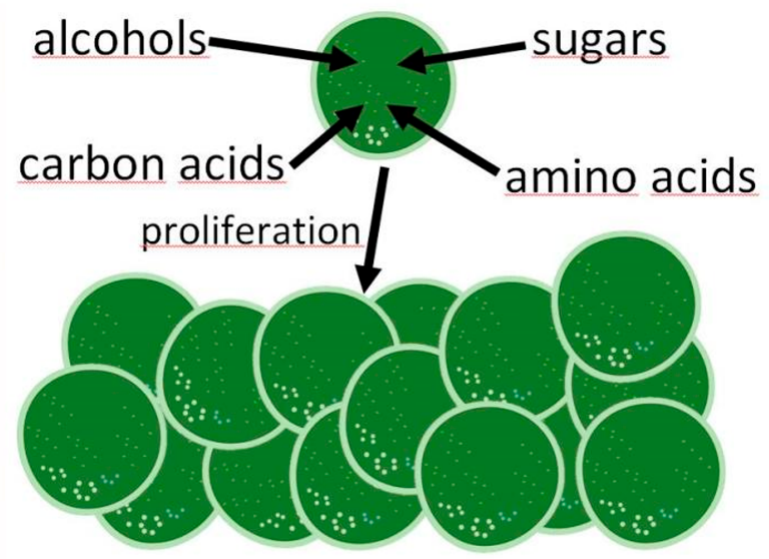

Cyanobacteria have been studied in recent decades to investigate the principle mechanisms of plant-type oxygenic photosynthesis, as they are the inventors of this process, and their cultivation and research is much easier compared to land plants. Nevertheless, many cyanobacterial strains possess the capacity for at least some forms of heterotrophic growth. This review demonstrates that cyanobacteria are much more than simple photoautotrophs, and their flexibility toward different environmental conditions has been underestimated in the past. It summarizes the strains capable of heterotrophy known by date structured by their phylogeny and lists the possible substrates for heterotrophy for each of them in a table in the Supporting Information. The conditions are discussed in detail that cause heterotrophic growth for each strain in order to allow for reproduction of the results. The review explains the importance of this knowledge for the use of new methods of cyanobacterial cultivation, which may be advantageous under certain conditions. It seeks to stimulate other researchers to identify new strains capable of heterotrophy that have not been known so far.

## 1 Introduction

Cyanobacteria have evolved to a photolithoautotrophic growth mode^1,2^ because they use light as an energy source (phototrophic), the inorganic water molecule as an electron source (lithotrophic) for NADPH production, and carbon dioxide, that has only one carbon atom, as the sole carbon source (autotrophic) (the different types of trophies and their possible combinations are explained in Table 1). This implies that autotrophic organisms must metabolically form all covalent bonds between carbon atoms. Unlike other phototrophic prokaryotes such as purple bacteria and green sulfur bacteria, which perform an anoxygenic mode of photosynthesis and have only one photosystem, cyanobacteria are defined as prokaryotes capable of oxygenic photosynthesis and possess two photosystems (I, for a review about photosystem I, please see ref (3), and II, for a review about photosystem II, please see ref (4)). Oxygenic photosynthesis requires two distinct photosystems to ensure sufficient reduction potential for water splitting (removal of electrons from the very electronegative oxygen and the release of protons and molecular dioxygen) on one hand and the production of enough ATP needed for carbon fixation in the Calvin cycle on the other. In eukaryotes such as algae and land plants, only oxygenic photosynthesis takes place, which consists of two photosystems. This fact is consistent with the endosymbiotic theory^5^ that the chloroplasts of algae and plants descended from cyanobacteria that invaded eukaryotic cells, the progenitor of modern algae and plants.

**Table 1:** Various Combinations of Trophies of Naturally Occurring Organisms

Growth mode	Energy source	Electron source	Carbon source
photolithoautotrophic	light	inorganic compounds (e.g., H_2_, Fe^2+^, NH_3_, H_2_S, H_2_O)	molecules consisting of only one carbon atom (e.g., CO_2_, HCOOH, CH_3_OH, CH_4_)
photolithoheterotrophic	light	inorganic compounds (e.g., H_2_, Fe^2+^, NH_3_, H_2_S, H_2_O)	organic molecules consisting of at least two carbon atoms (e.g., glucose, fructose, sucrose, glycerol, ...)
chemolithoautotrophic	inorganic compounds (e.g., H_2_, Fe^2+^, NH_3_, H_2_S)	inorganic compounds (e.g., H_2_, Fe^2+^, NH_3_, H_2_S)	molecules consisting of only one carbon atom (e.g., CO_2_, HCOOH, CH_3_OH, CH_4_)
chemolithoheterotrophic	inorganic compounds (e.g., H_2_, Fe^2+^, NH_3_, H_2_S)	inorganic compounds (e.g., H_2_, Fe^2+^, NH_3_, H_2_S)	organic molecules consisting of at least two carbon atoms (e.g., glucose, fructose, sucrose, glycerol, ...)
photoorganoheterotrophic	light	organic molecules consisting of at least two carbon atoms (e.g., glucose, fructose, sucrose, glycerol, ...)	organic molecules consisting of at least two carbon atoms (e.g., glucose, fructose, sucrose, glycerol, ...)
chemoorganoheterotrophic	organic molecules consisting of at least two carbon atoms (e.g., glucose, fructose, sucrose, glycerol, ...)	organic molecules consisting of at least two carbon atoms (e.g., glucose, fructose, sucrose, glycerol, ...)	organic molecules consisting of at least two carbon atoms (e.g., glucose, fructose, sucrose, glycerol, ...)
photomixotrophic	light	inorganic and organic molecules	molecules consisting of one carbon atom and molecules consisting of at least two carbon atoms

Due to their production of free molecular oxygen, cyanobacteria were among the first evolutionary organisms to protect themselves against radical oxygen species (ROS). As a result, cyanobacteria evolved aerobic respiration in addition to their oxygenic photosynthesis. The oxygen and organic molecules that have been formed in the light can be utilized for respiration in the dark. In contrast to eukaryotic algae and embryophytes, where these two processes are spatially separated in chloroplasts and mitochondria, they take place in the same compartment in cyanobacteria.^6^

**Figure 1:**
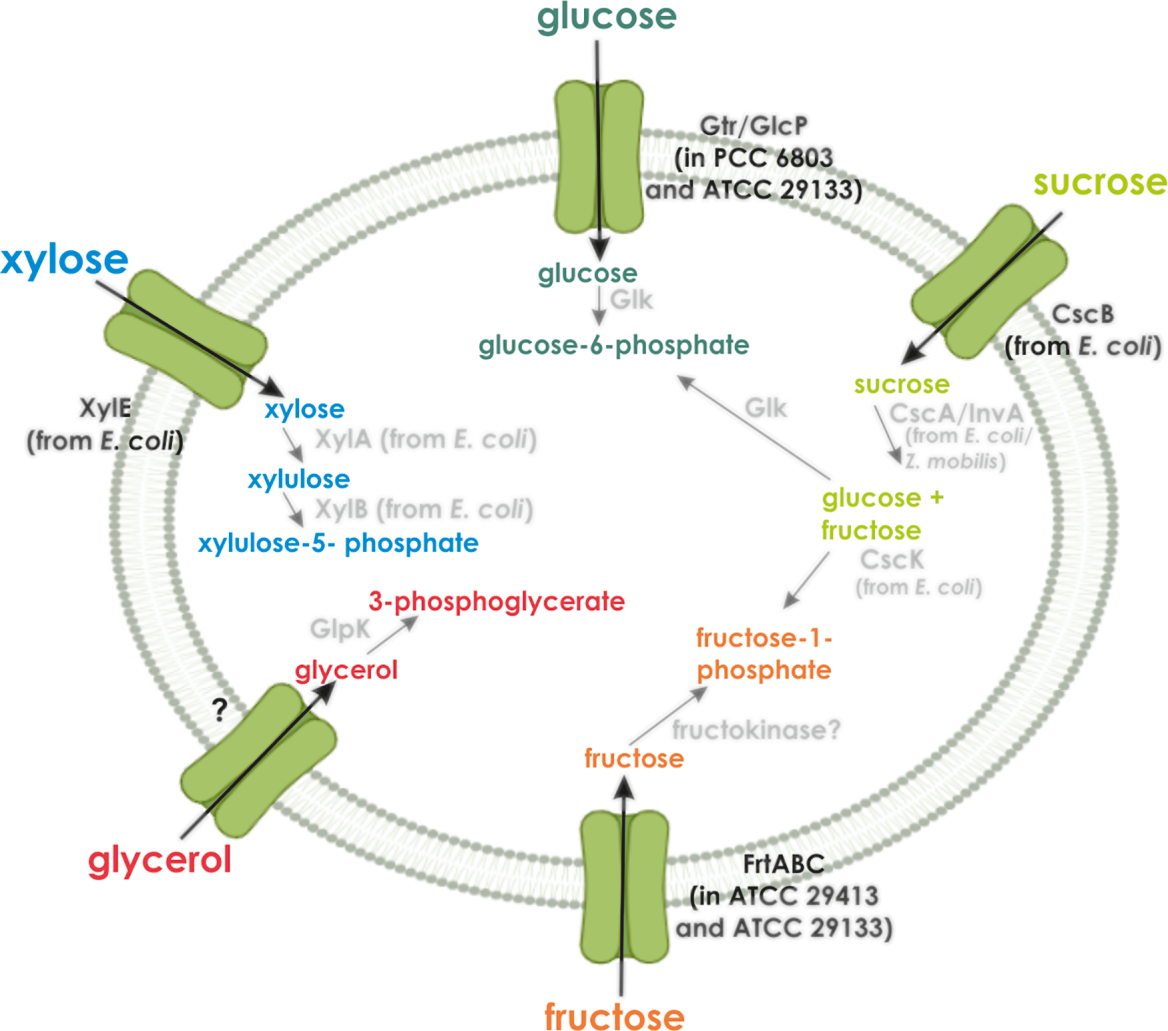
Overview of uptake and metabolization of diverse organic molecules by a cyanobacterial cell. GlpK, glycerol kinase; XylA, xylose isomerase; XylB, xylulokinase; Glk, glucokinase; CscA/InvA, invertase; CscK, fructokinase (from *E*. *coli*). The “?” at the glycerol transporter implies that this transporter and the corresponding gene in *Cyanothece* sp. ATCC 51142 have not been identified yet. The “?” after the (native cyanobacterial) fructokinase indicates that no gene within the genome of either *Anabaena* sp. ATCC 29413 or *Nostoc* sp. ATCC 29133 has been annotated as fructokinase so far.^24^

While all cyanobacteria, eukaryotic algae, and land plants can survive in periods of darkness by aerobic respiration, few strains of cyanobacteria^7−9^ and eukaryotic algae^10−12^ but no plants are able to grow in prolonged darkness, where carbon dioxide can no longer be fixed since NADPH and ATP production only occurs during the light reaction. In the dark, organic molecules are used as a source of carbon, energy, and electrons, and this growth mode is called chemoorganoheterotrophic growth (chemotrophic because energy is obtained through chemical reactions, organotrophic because the electrons are derived from organic molecules, and heterotrophic because organic substrates normally used for growth consist of at least two carbon atoms, as illustrated in Table 1). In the following sections, the terms photolithoautotrophic and chemorganoheterotrophic are abbreviated to photoautotrophic and chemoheterotrophic. Normally, cyanobacteria capable of chemoheterotrophic growth can use only one or two distinct organic molecules as a carbon source, and the substrates vary between cyanobacterial strains. The most prevalent substrates are sugars such as glucose, fructose, and sucrose and the alcohol glycerol.^7−9,13−23^ This is consistent with the fact that the genomes of many strains of cyanobacteria contain genes that are either responsible for the uptake of sugars or for their metabolism.^24^ Import through the outer membrane is more likely to occur through nonspecific porins,^25^ while for transport through the cytoplasmic membrane, homologues of the glucose transporter Glc in *Synechocystis* sp. PCC 6803^26^ and the fructose transporter FrtRABC in *Anabaena variabilis* ATCC 29413^27^ are widely distributed.^28^Figure 1 summarizes various substrates that are imported and metabolized by cyanobacteria.

*Synechocystis* sp. PCC 6803 is capable of a particular form of chemoheterotrophy, as it requires 5 min of daily illumination for glucose-dependent dark growth. Nevertheless, this growth mode can be considered as a kind of chemoheterotrophic growth since it is strictly dependent on the presence of glucose. This modified growth mode is termed light-activated heterotrophic growth (LAHG).^29^ A similar case is observed for *Synechococcus* sp. PCC 7002. This strain can grow in dim light (40 μW cm^–2^) in the presence of 55 mM glucose but not in total darkness at the same glucose concentration.^30^ Since a light intensity of 40 μW cm^–2^ itself does not induce (photoautotrophic) growth, it may be true (light-activated) heterotrophic growth.

Some strains of cyanobacteria are strictly light dependent but can also grow in the absence of photosystem II. If no water is split due to the lack of photosystem II, organic molecules serve as the electron source. Since photosystem I is still active, light can be considered as the energy source; however, the ATP originating from photosystem I is not sufficient for carbon dioxide fixation, and an organic electron source is additionally used for carbon assimilation. Therefore, this growth mode is termed photoorganoheterotrophic growth or, more briefly, photoheterotrophic growth.^8,9,23^ In cyanobacteria, photoheterotrophic conditions arise naturally through spontaneous mutations in genes encoding photosystem II subunits, but these mutations can also be engineered.^31−33^ Alternatively, photoheterotrophy can be induced by the herbicide 3-(3,4-dichlorophenyl)-1,1-dimethylurea (DCMU), which blocks electron transfer from photosystem II to the quinone pool.^34−37^

Some strains of cyanobacteria are unable to grow heterotrophically, so they cannot grow in the absence of light or either of their two photosystems; however, their photoautotrophic growth rate is further increased by the addition of organic molecules to their growth medium. This mode is called photomixotrophic growth. Although photomixotrophic growth is not a true form of heterotrophy, we will discuss it in this review because this growth mode is based on at least a partial growth dependence on organic molecules.

In all of the above cases, the appropriate substrate must be imported into the cell (for a review of the transport of organic substances across the cytoplamic membrane, please see ref (38)); however, strict photoautotrophy is not always caused by a lack of transport alone, as the entering molecule has to be metabolized in some way for the synthesis of ATP and NAD(P)H without accumulation of any toxic (by-)products.^39^

In this review, we summarize and analyze all available data on the different forms of heterotrophic (photomixotrophic, photoheterotrophic, or chemoheterotrophic) growth in cyanobacteria. In the past decades, there has been a strong focus on autotrophy, while little is known about facultative heterotrophy, as this topic has been rather neglected. The aim of this review is to expand knowledge about the possibility of heterotrophic cultivation of cyanobacterial strains that were previously considered strictly photoautotrophic in order to stimulate new research topics with cultivation forms that were previously not thought possible. Overall, the picture of cyanobacteria will change from purely photosynthetic organisms to highly flexible organisms that can adapt their metabolism to new circumstances when environmental conditions change.

## 2 Cyanobacterial Strains Capable of Heterotrophic Growth

The most comprehensive study of facultative photoheterotrophy among almost all cyanobacterial strains known at the time has been carried out by Rippka et al.^23^ Here we discuss cyanobacterial strains in alphabetical order following the sections of Rippka et al.^23^ since most of the physiological studies on chemoheterotrophy were performed decades ago; however, we slightly modified the system as we have combined sections IV and V since they form one common phylogenetic group.^40^ The strains described are mostly preserved in the Pasteur Culture Collection (PCC), the American Type Culture Collection (ATCC), the Culture Collection of Algae at the University of Texas (UTEX), or the Culture Collection of Algae and Protozoa (CCAP), and the underlying collection is part of the name of the strains (abbreviation followed by a number). Unless otherwise stated, all strains are cultivated in the BG11 medium (invented by Rippka et al.^23^), containing 0.18 mM K_2_HPO_4_·3H_2_O, 0.3 mM MgSO_4_·7H_2_O, 0.24 mM CaCl_2_·2H_2_O, 17.6 mM NaNO_3_, 2.8 μM Na_2_MgEDTA, 22.8 μM ferric ammonium citrate, 31 μM citric acid, 0.19 mM Na_2_CO_3_, 46 μM H_3_BO_3_, 1.6 μM Na_2_MoO_4_·2H_2_O, 9.7 μM MnCl_2_·4H_2_O, 0.77 μM ZnSO_4_·7H_2_O, 0.32 μM CuSO_4_·5H_2_O, and 0.17 μM Co(NO_3_)_2_·6H_2_O. Table S1 (in the Supporting Information) lists the strains known to date capable of heterotrophic growth and the organic substrate(s) used therefore, if known.

### 2.1 Section I: Chroococcales

Chroococcales are defined as unicellular cyanobacteria that divide by binary fission.^23^

#### 2.1.1 Chamaesiphon

PCC 7430 can grow photoheterotrophically on glucose, fructose, and sucrose.^23^

#### 2.1.2 Cyanothece (*Crocosphaera subtropica*)

Reddy et al.^41^ tested possible substrates for supporting photoheterotrophic growth of two *Cyanothece* sp. strains BH63 and BH68, which have been assigned by the ATCC as *Cyanothece* sp. strains ATCC 51141 and ATCC 51142. While glucose, fructose, and sucrose do not exert any beneficial effect, 10 mM glycerol allows photoheterotrophic growth with 20 μM DCMU in both the presence and absence of nitrate. Photoheterotrophic cultures in the absence of nitrate reach the stationary phase after 3 days, whereas they continue to grow at least for 6 days in the presence of nitrate.^41^ If the cells are incubated in diurnal 12 h light/12 h dark cycles, the nitrogenase was only active during the dark phase; however, even in continuous light its activity alternates in a 24 h cycle.^41,42^ In *Cyanothece* sp. ATCC 51142 the *nifHDK* genes encoding the subunits of the nitrogenase are exclusively expressed during the darkness in a 12 h light/12 h dark cycle or in regular intervals in a 24 h cycle when continuously illuminated. While the NifH subunit (Fe protein) is consequently degraded within this cycle regardless of illumination, the NifDK subunit (MoFe protein) is only completely degraded in 12 h light/12 h dark cycles but not in continuous light.^42^

The genome of *Cyanothece* sp. ATCC 51142 contains many genes that are regulated within a diurnal cycle^43,44^ and genes involved in glucose and pyruvate metabolism.^45^ Therefore, Feng et al.^46^ checked photomixotrophic growth of *Cyanothece* sp. ATCC 51142 on glucose, pyruvate, and glycerol under continuous illumination. In the absence of bound nitrogen, only 54 mM glycerol significantly increased the growth rate compared to photolithoautotrophic conditions, while the positive effect of 26 mM glucose can be neglected. Low concentrations of sodium pyruvate (9 mM) do not change the growth behavior for the first 6 days and then exhibit a deleterious effect, whereas higher concentrations (64 mM) cause a growth inhibition from the beginning. Glycerol doubles the growth rate of *Cyanothece* sp. ATCC 51142 in light both in the presence and in the absence of 18 mM sodium nitrate. The addition of 54 mM glycerol or 26 mM glucose increases the nitrogen-dependent hydrogen production during the exponential growth phase in continuous light by a factor of 5.0 and 2.6, respectively. 11 mM sodium pyruvate on the other hand slightly reduces the hydrogen production by a factor of 0.8.^46^

Isotope (^13^C) labeling reveals that under mixotrophic conditions alanine, histidine, and serine are exclusively built from glycerol as the only carbon source. The authors therefore call it photoheterotrophic metabolism since carbon dioxide does not contribute to the synthesis of the three amino acids in this experiment; however, we consider it to be photomixotrophic growth, as PSII has not been blocked (e.g., by DCMU). In the absence of sodium nitrate the portion of carbon derived from glycerol within alanine, histidine, and serine is dramatically reduced. Interestingly glucose and pyruvate also contribute to the formation of amino acids (i.e., they are metabolized within the cell), although they do not significantly increase the growth rate, although the substance does not support growth.^46^ Recently the *Cyanothece* sp. ATCC 51142 has been renamed to *Crososphaera subtropica* ATCC 51142.^47^

#### 2.1.3 Gloeocapsa

*Gloeocapsa* sp. strain PCC 7428 grows photoheterotrophically on glucose, fructose, ribose, and sucrose, whereas *Gloeocapsa* sp. strain PCC 7501 can only do so on glucose.^23^

#### 2.1.4 Synechococcus

For a review of photomixotrophic growth of marine *Synechococcus* see Munoz-Marin et al.^48^ Duhamel et al.^49^ studied photomixotrophic metabolism in marine undefined *Synechococcus* sp. strains that (along with *Prochlorococcus* sp.) contribute to most of the marine photosynthesis.^50−52^*Synechococcus* sp. assimilates glucose, leucine, and ATP at low rates and molecules containing nitrogen and phosphorus at a higher rate. Molecular assimilation is higher in light under photomixotrophic conditions than in the dark under heterotrophic conditions or when photosystem II is inhibited.^49^

*Synechococcus* sp. PCC 7002 (designated *Agmenellum quadruplicatum* PR-6 in the publication by Van Baalen^53^) grows (light activated) heterotrophically in the presence of 44–55 mM (and to a lesser extent also at 11 mM) glucose, if weak light (40 μW cm^–2^) is present; however, no growth occurs at the same glucose concentration in total darkness. Under high light conditions that allow for normal photoautotrophic growth, glucose exerts no stimulating effect. As a result, the contribution of carbon derived from glucose incorporated into amino acids is higher under dim light than under strong light. Acetate fails to induce the heterotrophic growth of *Agmenellum quadruplicatum* PR-6 in dim light.^30^ In addition, *Synechococcus* sp. PCC 7002 grows photoheterotrophically in the presence of DCMU on glycerol and to a lesser extent also on glucose.^23^ Lambert and Stevens^54^ have also tested several substrates for photoheterotrophic growth. Fructose, sodium malate, sodium citrate, and glucose fail in this study (both at 10 and 50 mM), while growth occurs on heavily inocculated plates supplemented with glycerol (1–100 mM) and DCMU. Nevertheless, the pigmentation of the cells is reduced at 10 mM or higher concentrations of glycerol; however, dark green cells with normal pigments have formed on plates supplemented with 30 or 100 mM glycerol. This glycerol-tolerant strain named *Agmenellum qudruplicatum* PR-6G2 does not differ much under photoautotrophic or photomixotrophic conditions. PR-6 and PR-6G2 grow at generation times of 4.8 and 4.7 h (photoautotrophic conditions), 4.9 and 5.2 h (photomixotrophic conditions with 1 mM glycerol), and 4.6 and 4.8 h (photomixotrophic conditions with 30 mM glycerol), respectively. These data show that glycerol exerts an inhibitory effect on both strains at low concentrations and little inhibition on PR-6G2 at high concentrations, while for PR-6 high concentrations of glycerol stimulate the growth rate. Interestingly, strain PR-6 but not the glycerol-tolerant strain PR-6G2 can use glycerol for photomixotrophic growth. However, strain PR-6G2 grows under photoheterotrophic conditions (10 μM DCMU, 1–30 mM glycerol) and faster in the presence of high glycerol concentration. The generation times of PR-6 and PR-6G2 are 19.6 and 17.8 h at 1 mM glycerol and 17.8 and 12.2 h at 30 mM glycerol, respectively.^54^ Rippka et al.^23^ have identified some other *Synechococcus* sp. strains capable of facultative photoheterotrophy. *Synechococcus* sp. PCC 7511 can grow on both glucose and sucrose. The strains *Synechococcus* sp. PCC 7003 (*Coccochloris elabens* 17a,^55^) and PCC 73109 (*Agmenellum quadruplicatum* BG-1,^53^) grow on glycerol and weakly on glucose, while the latter strain additionally exhibits weak growth on fructose. *Synechococcus* sp. PCC 7335 can only grow on fructose.^23^

**Figure 2:**
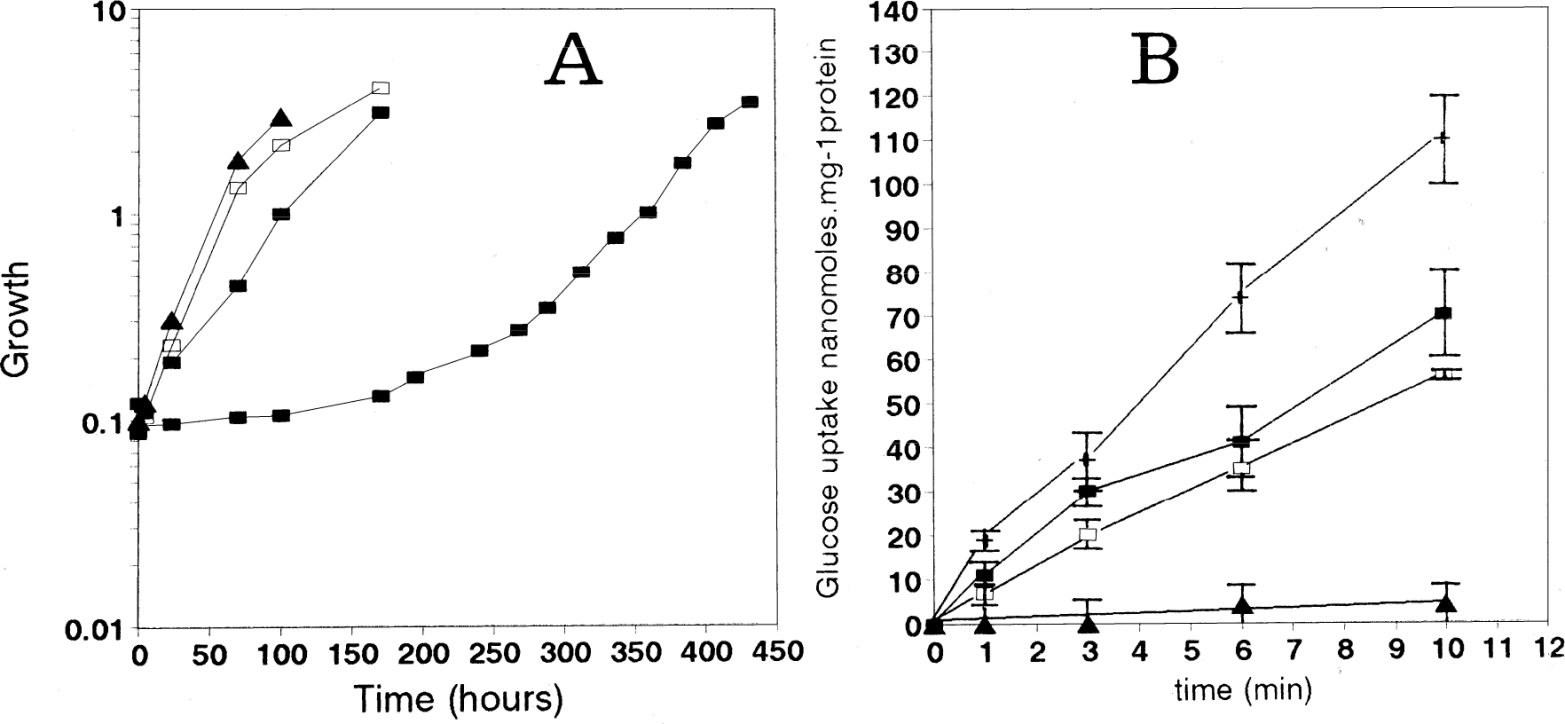
Growth (A) and glucose uptake (B) of *Synechococcus* PCC7942 WT and R2–1 cells incubated under the indicated trophic conditions. All cultures of R2–1 contained streptomycin. A: WT, photoautotrophy (▲); R2–1, photoautotrophy (□), transferred from photoautotrophy to photoheterotrophy (■, lower line), maintained under photoheterotrophy (■, upper line). B: WT (▲); R2–1 pregrown under photoautotrophy (□), adapted to photoheterotrophy (■). WT *Synechocystis* PCC6803 grown under photoautotrophy (+). The growth curves shown represent one typical set of 3–4 repeats. Reprinted with permission from ref (39). Copyright 1998 Oxford University Press.

Kratz and Myers^56^ observed an increased respiration in *Synechococcus* sp. PCC 6301 (named *Anacystis nidulans* in this publication) in the dark in the presence of 100 mM glucose or 100 mM fructose, although this organism and its closely related strain *Synechococcus* sp. PCC 7942^57^ have been designated as strictly photoautotrophic.^23^ When Zhang et al.^39^ introduced the *Synechocystis* sp. PCC 6803 *gtr* gene coding for glucose permease^26^ (Figure 1) on an autonomously replicating plasmid, the transgenic strain *Synechococcus* sp. PCC 7942 *gtr*^+^ becomes capable of photoheterotrophic growth in the presence of 56 mM glucose (Figure 2), but the plasmid cannot be maintained stably. In contrast, the integration of *gtr* into the chromosome results in a strain that can no longer grow on 56 mM glucose. Niederholtmeyer et al.^58^ introduced the *glf* gene from *Zymomonas mobilis*,^59^ which encodes a glucose- and fructose-facilitated diffusion transporter into *Synechococcus* sp. PCC 7942, and the new transgenic strain can grow in the dark on both 500 μM glucose and 500 μM fructose.

**Figure 3:**
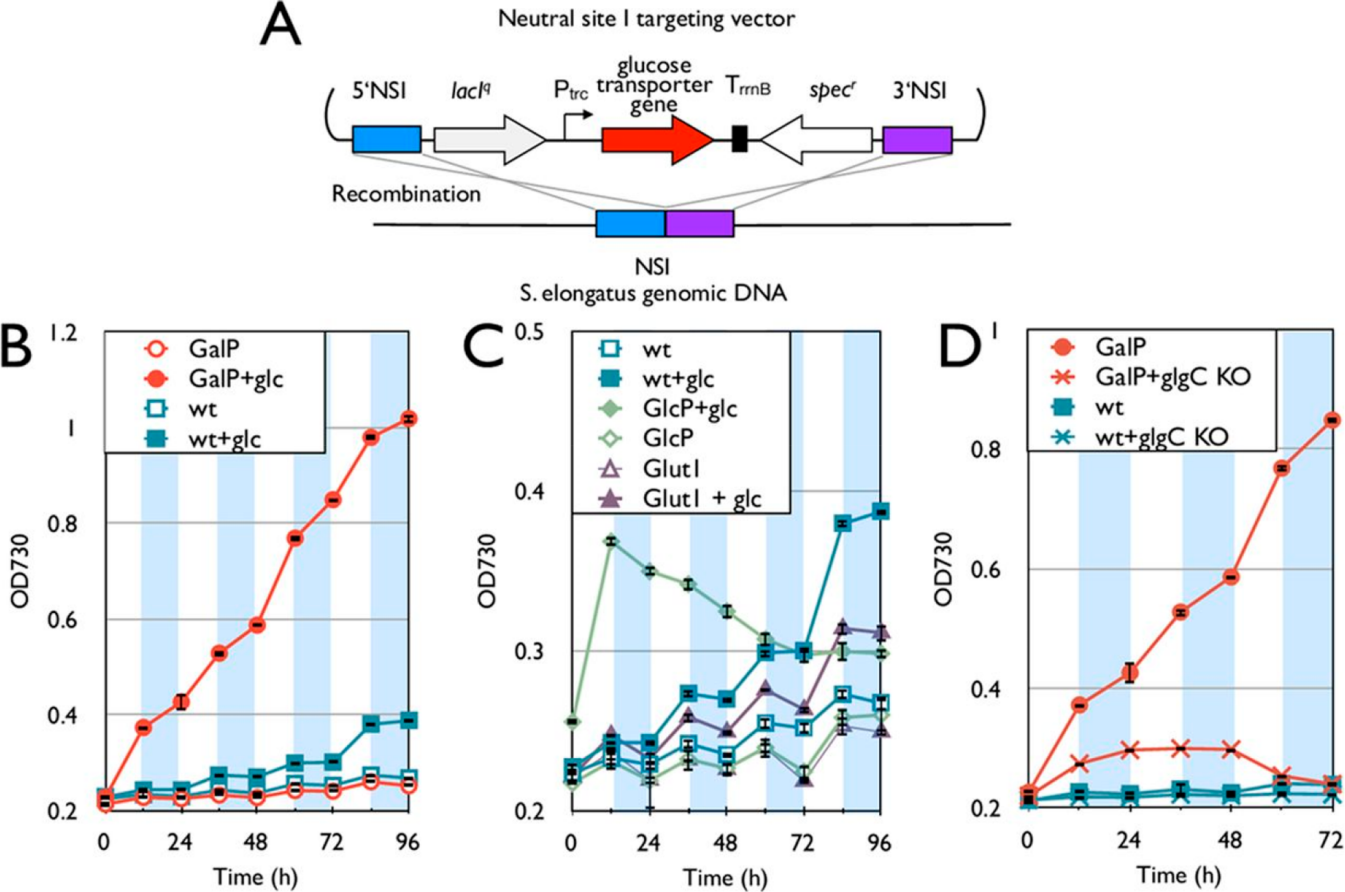
Installation of the glucose transporter to *S*. *elongatus*. (A) Schematic representation of the glucose transporter gene integration into the *S*. *elongatus* genome. (B) Growth curve of the *galP* strain (red) and wild type (blue) with and without 5 g/L glucose. (C) Growth curve of the *Glut1* (purple) and *glcP* (green) strains and the wild type. (D) Growth curve of the *galP* strain and the wild type with and without *glgC* deletion (glgC KO). For panels B, C, and D, empty symbols are samples in BG-11 medium without glucose, while solid symbols indicate results with BG-11 containing 5 g/L glucose. White and shaded areas indicate the light and dark cycles, respectively. All *y* axes denote OD_730_, although the scales differ for visibility. Error bars represent standard deviations (in triplicate). NSI, neutral site 1. Reprinted with permission from ref (60). Copyright 2013 American Society for Microbiology.

**Figure 4:**
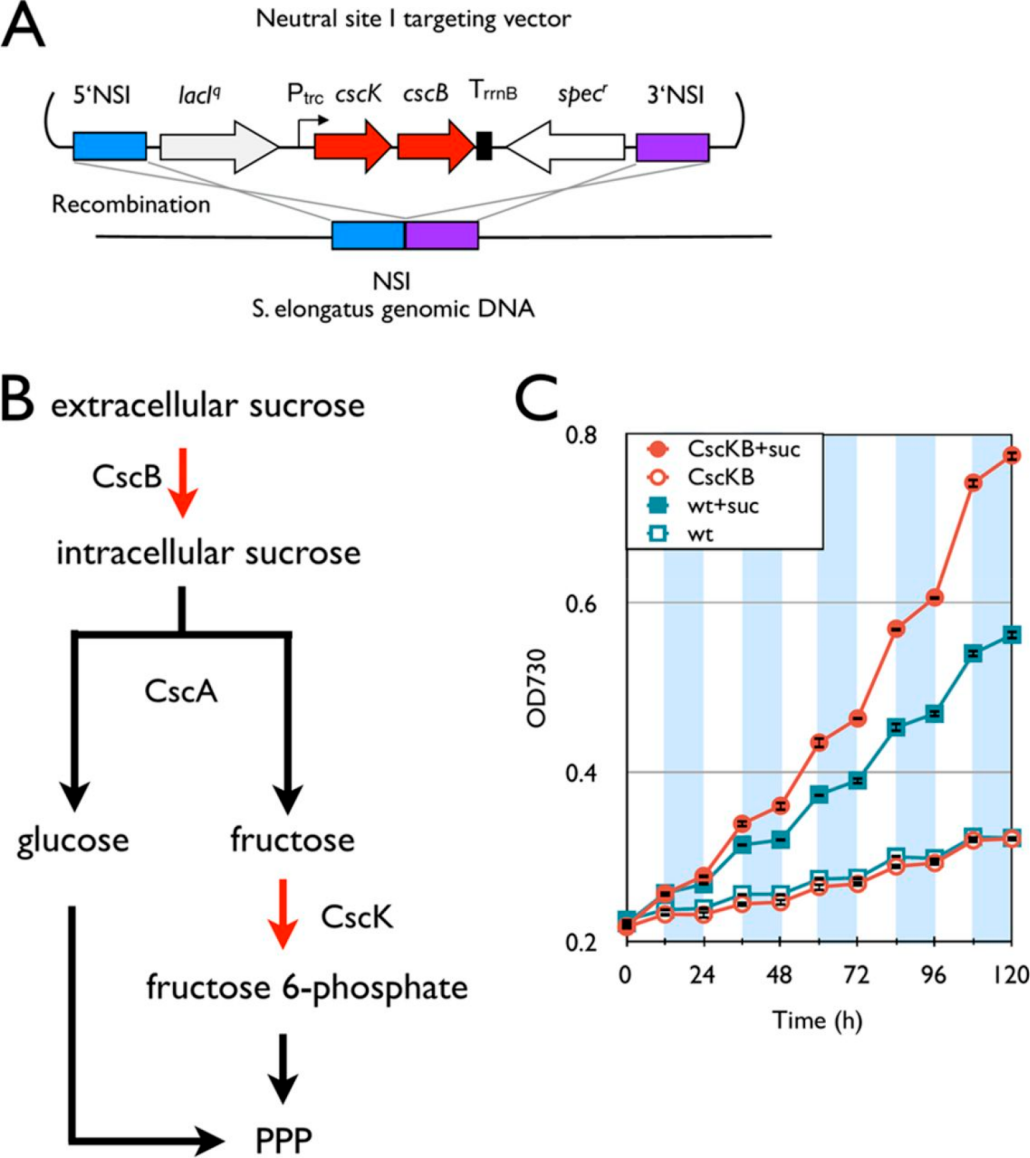
Installation of the sucrose degradation pathway. (A) Schematic representation of integration of the sucrose degradation pathway into the *S*. *elongatus* chromosome. (B) Synthetic sucrose degradation pathway in *S*. *elongatus*. Red arrows indicate steps catalyzed by heterologous enzymes. PPP, pentose phosphate pathway. (C) Growth curves of the *cscB*–*cscK* strain (red) and the wild type (blue). Empty and filled symbols indicate growth without and with 5 g/L sucrose, respectively. White and shaded areas indicate light and dark cycles, respectively. Error bars represent standard deviations (in triplicate). Reprinted with permission from ref (60). Copyright 2013 American Society for Microbiology.

**Figure 5:**
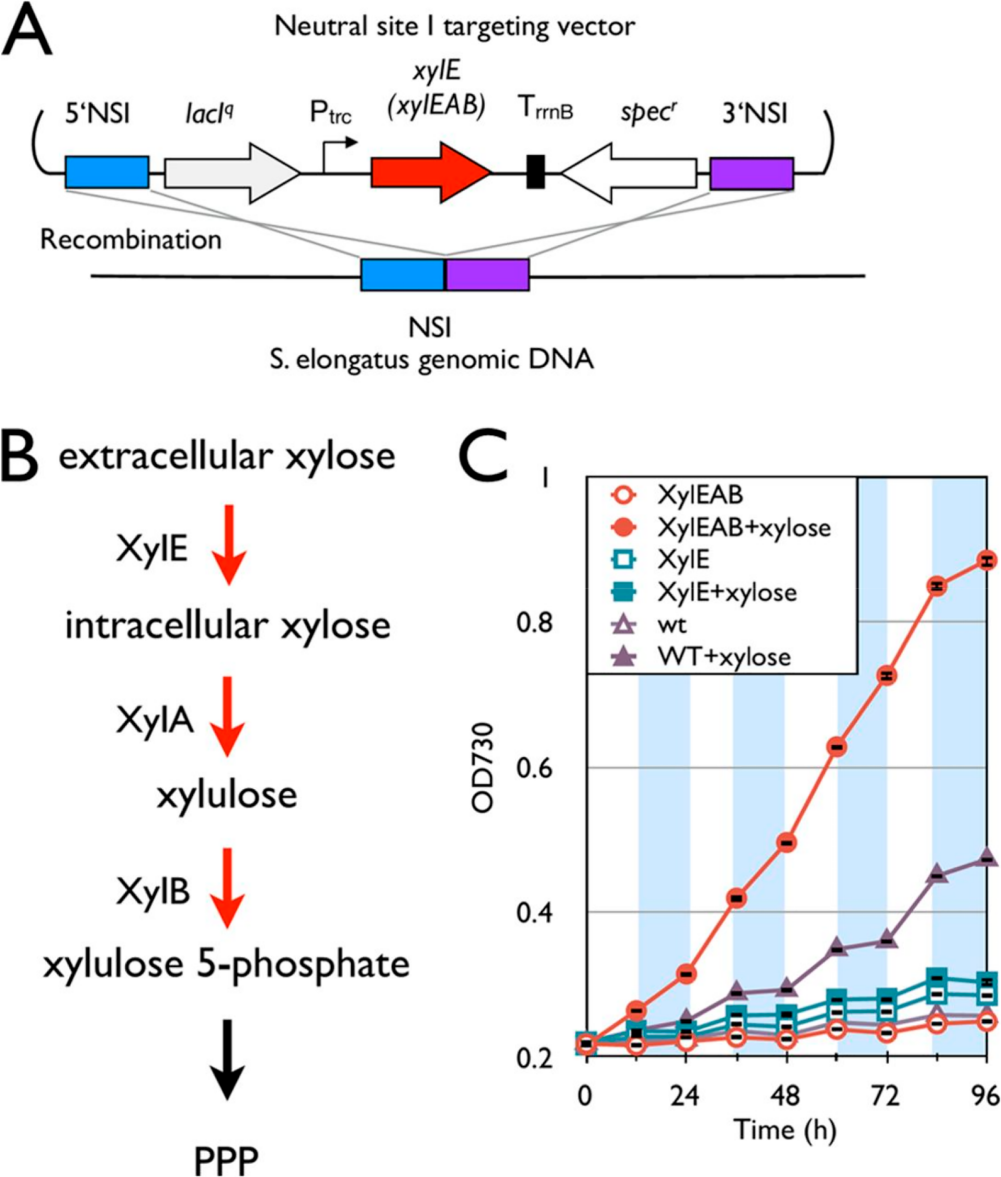
Installation of the xylose degradation pathway. (A) Schematic representation of integration of the xylose degradation pathway into the *S*. *elongatus* chromosome. (B) Synthetic xylose degradation pathway in *S*. *elongatus*. Red arrows indicate steps catalyzed by heterologous enzymes. (C) Growth curves of the *xylE* and *xylEAB* strains and wild type. Empty and filled symbols indicate growth without and with 5 g/L xylose, respectively. White and shaded areas indicate light and dark cycles. Error bars represent standard deviations (in triplicate). Reprinted with permission from ref (60). Copyright 2013 American Society for Microbiology.

McEwen et al.^60^ investigated the ability of *Synechococcus* sp. PCC 7942 to grow on different substrates and find that glucose, sucrose, and xylose slightly increase growth under 12 h light/12 h dark cycles. In order to increase growth on the sugars mentioned above, various genes from other organisms that encode sugar transporters or enzymes involved in sugar metabolism are introduced. All experiments by McEwen et al.^60^ were performed in BG11 medium.^23^ While acquisition of *Glut1* from human erythrocytes^61^ and *gtr* from *Synechocystis* sp. PCC 6803^26^ reduce diurnal growth on 28 mM glucose compared to the wild type (Figure 3C), introduction of *galP* from *E*. *coli*,^62^ which encodes the glucose/galactose permease, leads to enormously increased growth at the same glucose concentration (Figure 3B). Transfer of *cscB* and *cscK* (Figure 1) from *E*. *coli*, which encode a sucrose transporter and a fructokinase (Figure 4B), significantly enhances growth on 15 mM sucrose under 12 h light/12 h dark conditions (Figure 4C). The introduction of only *xylE* (Figure 1) from *E*. *coli*, which encodes a xylose transporter, does not only increase diurnal growth on 28 mM xylose but also abolishes any growth-stimulating effect of this sugar (Figure 5C). However, the cotransfer of *xylA* and *xylB* (Figure 1) from *E*. *coli*, which code for a xylose isomerase and a xylulokinase (Figure 5B), together with *xylE* on an operon, leads to a greatly increased growth on 28 mM xylose under 12 h light/12 h dark conditions compared to the wild type (Figure 5C). It is assumed that the inability of xylose to enhance the growth of transgenic PCC 7942 *xylE*^+^ is caused by an accumulation of this sugar inside the cell due to its slow metabolization when genes such as *xylA* and *xylB*, which are important for xylose metabolism, are absent.^60^ In contrast to *Synechocystis* sp. PCC 6803,^63^ the acquisition of *xylAB* alone without *xylE* in *Synechococcus* sp. PCC 7942 has not yet been tested. While Zhang et al.^39^ reported a toxic effect of glucose when *gtr* was integrated into the chromosome, McEwen et al.^60^ observed rapid growth (factor 1.5) within the first 12 h but then a decrease until 72 h with subsequent stagnation. Despite this, the cell density is still higher than that of cultures of the same strain in the absence of glucose. However, the effect of glucose on *Synechococcus* sp. PCC 7942 (wild type and all transgenic mutant strains created by Zhang et al.^39^) was not tested for more than 96 h by McEwen et al.^60^ Besides Zhang et al.^39^ used the 2-fold glucose concentration for their experiments compared to McEwen et al.^60^ (56 vs 28 mM).

#### 2.1.5 Synechocystis

Rippka et al.^23^ identified several *Synechocystis* sp. strains capable of photoheterotrophic growth dependent on glucose. *Synechocystis* sp. PCC 6702 (*Aphanocapsa* HD,^55^), PCC 6805 (*Aphanocapsa* sp.,^55^), PCC 6806 (*Aphanocapsa* sp.,^55^), PCC 6905, and PCC 7201 can only grow on glucose. PCC 7509 also grows on sucrose, and PCC 6906 (also *Eucapsis* sp.) can grow well on glycerol but only weakly on glucose.^23^*Synechocystis* sp. PCC 6714 (*Aphanocapsa* sp.,^55^) can grow both photoheterotrophically^8,23^ and chemoheterotrophically^64^ dependent on glucose in the BG11 medium.^23^ The closely related strain, *Synechocystis* sp. PCC 6803 (*Aphanocapsa* sp.,^55^), also grows on 28 mM glucose under photoheterotrophic conditions,^8,23,31^ but contradicting results have been published for its growth in the dark. Rippka^8^ states that no chemoheterotrophic growth on glucose was observed. However, four later publications report the chemoheterotrophic growth of this strain under different glucose concentrations: Astier et al.,^31^ 111 mM, generation time: 19 h; Labarre et al.,^65^ 56 mM, generation time: 22 h; Joset et al.,^66^ 5 mM, generation time: 25 h; Jeanjean et al.,^67^ 56 mM, generation time: 24 h. Under photomixotrophic conditions, 10 mM glucose is slightly toxic for *Synechocystis* sp. PCC 6803.^68^ However, Williams^69^ isolated a glucose-tolerant variant of this strain, which has since become the dominant variant for research, particularly when glucose is added to the growth medium.

Anderson and McIntosh^29^ discovered that a glucose-tolerant strain of *Synechocystis* sp. PCC 6803^69^ grows on glucose in the dark, when cells receive 5 min of daily illumination (white light, 40 μmol m^–2^ s^–1^). Since glucose is necessary and the growth rate depends on its concentration, this growth mode is named light-activated heterotrophic growth (LAHG). The different results obtained in the past on chemoheterotrophic growth are probably due to the fact that some scientists have inadvertently interrupted the darkness when measuring cell density, while others have maintained strict darkness. Anderson and McIntosh^29^ tested different media for their experiments, including BG11^23^ with 5 mM TES pH = 8.0 supplemented with 5 mM glucose and BG11 supplemented with 56 mM glucose and 6 mM *N*-2-hydroxyethylpiperazine-*N*′-2-ethanesulfonic acid (HEPES) at pH 7.5. The latter medium has been reported to be useful for photoheterotrophic growth.^65^ LAHG is improved with the latter medium, and even slow growth occurs within the first 6 days under these conditions; however, a decline is noted thereafter. LAHG has also been observed in a mutant strain lacking all three *psbA* genes and thus a functional photosystem II.^29,32^ Later, Pils et al.^70^ discover that a functional *coxBAC* locus encoding an *aa3* type cytochrome *c* oxidase (homologous to the cytochrome *c* oxidase in mitochondria) is necessary for the LAHG of *Synechocystis* sp. since a *coxBAC* deletion mutant strain loses the capacity of LAHG.

The *cytM* gene, which encodes a cytochrome *c*-type protein, significantly affects heterotrophic growth, for which no good explanation currently exists. Hiraide et al.^71^ investigated the capacity of a *Synechocystis* sp. PCC 6803 *cytM* knock out mutant strain under different growth modes and observed faster growth compared to the wild type on 5 mM glucose in both the light and the dark when illuminated for 15 min per day. Furthermore, *Synechocystis* sp. PCC 6803 *cytM*^–^ can grow in permanent darkness with a long generation time of 33 h. All the experiments were performed in BG11 medium^23^ buffered with 20 mM HEPES–KOH pH = 8.2 and supplemented with 5 mM glucose as needed.^65^

Lopo et al.^72^ studied the differential growth and behavior of *Synechocystis* sp. PCC 6803 in terms of temperature, pH, trophic mode, glucose, and nitrate concentrations. In these experiments, the presence (photomixotrophy) or absence (photoautotrophy) of glucose causes a significant difference in growth behavior. The growth of autotrophic cultures is largely influenced by pH and temperature, and higher growth is observed at higher pH. On the other hand, photomixotrophic cultures were fairly stable to these changes, although contrary to photomixotrophic cultures, they even grow slightly faster at lower pH.

**Figure 6:**
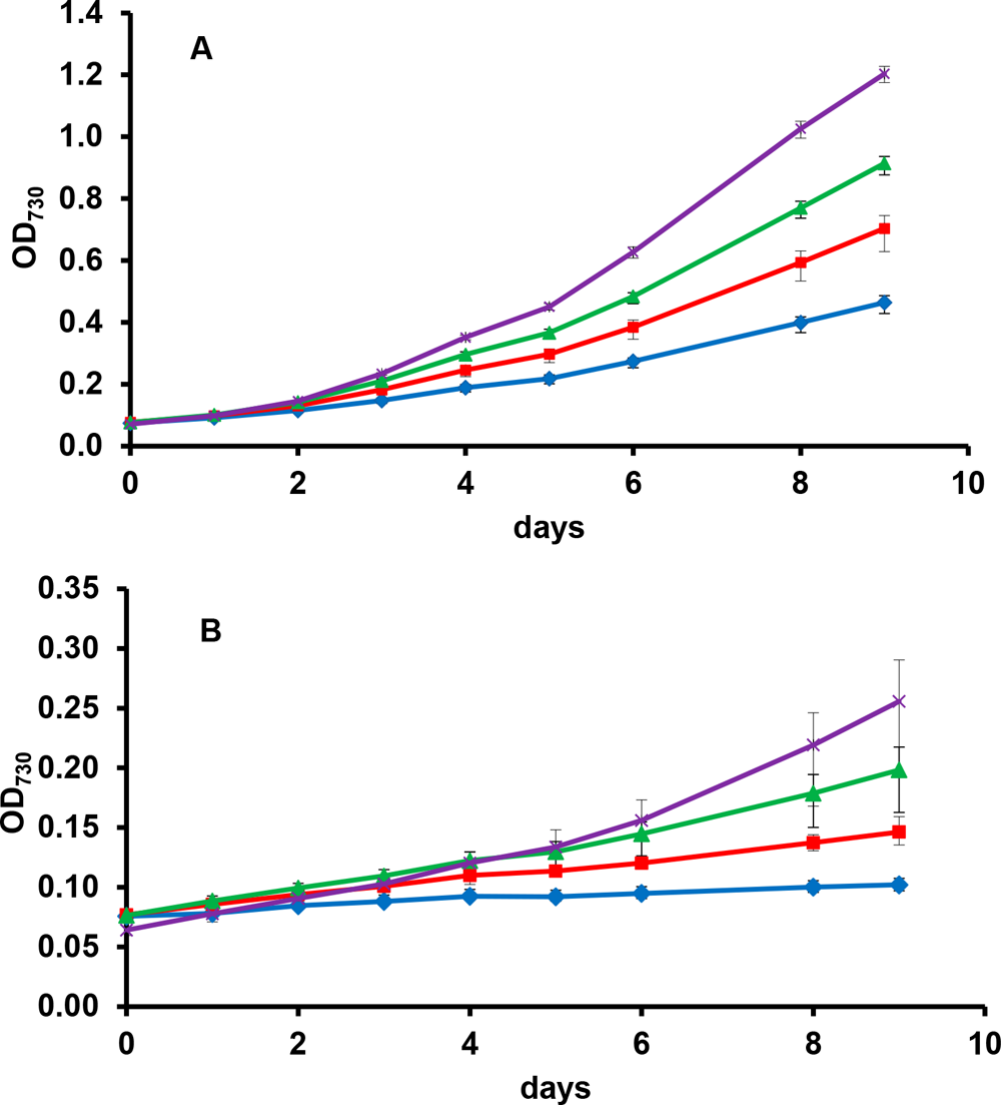
Photomixotrophic (A) and photoheterotrophic (B) growths of *Synechocystis* sp. PCC 6803 *gtr*^–^ dependent on (blue diamond) 0 mM, (red square) 50 mM, (green triangle) 100 mM, and (×) 200 mM fructose. Reprinted with permission from the authors of ref (74).

Both *Synechocystis* sp. strains PCC 6714 and PCC 6803 are highly sensitive to fructose,^66,68,73^ and *Synechocystis* sp. PCC 6803 is killed even at a concentration of 10 mM fructose.^68^ The mechanism of fructose toxicity is not well understood; however, both glucose and fructose enter the cell through the same mechanisms because mutating the gene *gtr*, which encodes the glucose permease^26^ (Figure 1), abolishes the toxicity of fructose.^68^ Since *Synechocystis* sp. PCC 6803 *gtr*^–^ can no longer use glucose for photoheterotrophic growth^68^ this mutant strain is considered strictly photoautotrophic. Stebegg et al.^74^ discovered that enormously high concentrations (50–200 mM) of fructose not only are harmless but can also support both photomixotrophic (Figure 6A) and photoheterotrophic growth (Figure 6B) of *Synechocystis* sp. PCC 6803 *gtr*^–^, while glucose, applied at the same concentration, does not.

Recently Rapp et al.^75^ investigated the effect of 7-deoxysedoheptulose (7dSh) on several cyanobacteria. 7dSh is excreted by *Synechococcus* sp. PCC 7942 (and by noncyanobacterial strains like *Streptomyces setonensis*) to inhibit the 3-dehydroquinate synthase (DHQS) of other organisms (including cyanobacteria), thereby preventing the growth of organisms of the same ecological niche. 7dSh is imported by *Synechocystis* sp. PCC 6803 via the Gtr permease since a spontaneous mutant strain resistant to 7dSh (at a concentration of 250 μM) contains a single-point mutation in the *gtr* gene. This strain has also lost the ability to grow on glucose as well as sensitivity to fructose. An artificially generated mutant strain whose *gtr* gene is replaced by a spectinomycin resistance cassette shows the same behavior toward 7dSh. In contrast to fructose, the toxicity of 7dSh is not alleviated by delivery of a 20-fold glucose concentration (5 mM).^75^

Lee et al.^63^ introduced the *xylA* and *xylB* genes encoding a xylose isomerase and a xylulokinase (Figure 1), respectively, on an operon from *E*. *coli* into *Synechocystis* sp. PCC 6803, resulting in a strain capable of growth on xylose. When *xylAB* is transferred into an ethylene-producing mutant strain of *Synechocystis* sp. PCC 6803, addition of xylose further increases the production of ethylene.^63^ Finally, the introduction of *xylAB* into a strain whose *glgC* gene responsible for glycogen synthesis has been deleted^76,77^ results in higher keto acid production.^63^ In the same year, Xiong et al.^78^ report that this *glgC*^–^/*xylAB*^+^ mutant strain^63^ metabolizes xylose to acetate in the absence of bound nitrogen. Deletion of the *slr0453* gene, thought to encode a phosphoketolase, decreases acetate production in the light and eliminates it in the dark, demonstrating the importance of the phosphoketolase signaling pathway for heterotrophy in cyanobacteria.^78^

#### 2.1.6 *Thermosynechococcus elongatus*

Zilliges and Dau^79^ investigated different mono- and disaccharides for their potential to induce photomixotrophic or photoheterotrophic growth of *Themosynechococcus elongatus*. 50 mM glucose and 50 mM fructose and to a lesser extent 50 mM galactose, 35 mM lactose, and 35 mM sucrose act as substrates, while 75 mM arabinose, 35 mM maltose, and 35 mM trehalose do not significantly affect growth. 75 mM xylose even shows an inhibitory effect. Although 50 mM fructose is beneficial initially, it causes cell bleaching after 4 days. 50 mM fructose also supports chemoheterotrophic growth in total darkness or LAHG (at a daily illumination of 15 min).

### 2.2 Section II: Pleurocapsales

Pleurocapsales are defined as unicellular cyanobacteria that divide by multiple fission.^23^*Chroococcidiopsis* is listed within the filamentous cyanobacteria due to phylogenetic reasons.^40^

#### 2.2.1 *Dermocarpa*

*Dermocarpa* sp. PCC 7301 (also *Dermocarpa violacea* in CCAP) can grow photoheterotrophically on glucose, fructose, and sucrose, while *Dermocarpa* sp. strains PCC 7304 and PCC 7437 (also *Chroococcidiopsis cyanoasphera* strain 1965/25^80^) can only use glucose and sucrose for heterotrophy, and *Dermocarpa* sp. PCC 7438 (also *Chroococcidiopsis cyanosphaera* strain 1965/26^80^) grows on glucose and fructose but not on sucrose.^23^

#### 2.2.2 *Dermocarpella*

*Dermocarpella* sp. PCC 7326 can grow heterotrophically on glucose, fructose, and sucrose.^23^

#### 2.2.3 *Myxosarcina*

*Myxosarcina* sp. strain PCC 7312 grows photoheterotrophically on glucose, while strain PCC 7325 is able to grow on glucose and sucrose. *Myxosarcina chroococcoides* CCAP 1451/1 (named *Chroococcidiopsis* sp. PCC 7203 in ref (23)) grows photoheterotrophically on glucose, fructose, and sucrose.^23^

#### 2.2.4 *Xenococcus*

*Xenococcus* sp. PCC 7307 grows photoheterotrophically on glucose and sucrose, while *Xenococcus* sp. strains 7305 and 7306 do not.^23^

#### 2.2.5 *Pleurocapsa* Group

This group consists of several strains within section II that cannot be assigned to any of the five genera mentioned. PCC 7310, PCC 7314, PCC 7319, PCC 7320, PCC 7320, PCC 7321, PCC 7322, PCC 7327 (also *Pleurocapsa minor* OH-69-pm^80^), and PCC 7516 (also *Hyella caespitosa*(80)) can grow photoheterotrophically on sucrose, while PCC 7314, PCC 7319, PCC 7320, and PCC 7322 grow photoheterotrophically on fructose. PCC 7317, PCC 7320, PCC 7322, and PCC 7506 grow photoheterotrophically on glucose.^23^

### 2.3 Section III: Oscillatoriales

Oscillatoriales are defined as filamentous cyanobacteria that do not form heterocysts in the absence of bound nitrogen.^23^

#### 2.3.1 *Arthospira platensis*

Phycocyanin is produced in bioreactors from batch cultures of *Arthospira platensis*. Zhang et al.^81^ show that the addition of 11 mM glucose to a batch culture of *Arthospira platensis* (called *Spirulina platensis* by Zhang et al.^81^) in a growth medium according to Zarouk^82^ increases cell density and produced phycocyanin by a factor of 4.29 and 3.05, respectively.

#### 2.3.2 *Lyngbya*

*Lyngbya lagerheimii* strain Montauk grows heterotrophically in the presence of 55 mM glucose under dim light (40 μW cm^–2^), which does not allow autotrophic growth. In addition very little growth is observed on glucose in the dark. In contrast, at high light intensities that allow for autotrophic growth, glucose does not exert a growth-stimulating effect. Consequently, when *Lyngbya lagerheimii* is cultured under low light, the glucose taken up from the external medium contributes more to the cell’s own amino acids compared with high light conditions. While sucrose also supports slight heterotrophic growth in low light, formate, acetate, pyruvate, and succinate do not.^30^ Rippka et al.^23^ tested the photoheterotrophic growth capacity (with DCMU) of *Lyngbya lagerheimii* and identified glucose, fructose, and sucrose as possible substrates (the strain is named PCC 7104 and placed into the LPP group by Rippka et al.^23^), while *Lyngbya* sp. PCC 7419 (placed into the LPP group by Rippka et al.^23^) grew only weakly on glucose.^23^

#### 2.3.3 LPP strains

This group was created by Rippka et al.^23^ and includes the genera *Lyngbya*, *Phormidium*, and *Plectonema*. Many strains within the LPP group can use more than one particular organic compound as a carbon source.^23^ In this paragraph, the strains that have not been assigned to any genus in 1979 are listed. Those strains capable of heterotrophy, and classified by Rippka et al.^23^ as LPP strains but previously known by a different generic name, are discussed under their former name in this review. PCC 7410 and PCC 7505 grow photoheterotrophically on glucose, fructose, ribose, and sucrose. PCC 7123 grows on glucose, fructose, and sucrose. PCC 7114 grows on glucose and sucrose, while PCC 7113 grows photoheterotrophically only on sucrose.^23^

#### 2.3.4 *Microcoleus*

*Microcoleus vaginatus* (named LPP PCC 7407 in ref (23)) grows on glucose, fructose, and ribose.^23^

#### 2.3.5 *Oscillatoria*

Rippka et al.^23^ analyzed several *Oscillatoria* strains for their facultative photoheterotrophy. PCC 6602 and PCC 7515 grow on glucose and fructose. PCC 6412 (also *Lyngbya* sp. UTEX 1546) also grows on glucose and fructose but only weakly, while PCC 6407 and PCC 6506 (also *Lyngbya kuetzingii* UTEX 1547) grow weakly on glucose.^23^*Oscillatoria williamsii* MEV (LPP strain PCC 7105 in ref (23)) grows on glycerol and weakly on glucose, fructose, and sucrose.^23^*Oscillatoria okeni* TISTR 8549 produces poly(3-hydroxybutyrate-*co*-3-hydroxyvalerate) (PHBV) in the absence of bound nitrogen. Addition of acetate to the growth medium increases PHBV production by *Oscillatoria okeni* TISTR 8549 under chemoheterotrophic conditions in the dark but not in the presence of light under photomixotrophic conditions.^83^

#### 2.3.6 *Phormidium*

*Phormidium* sp. UTEX 1540^84^ grows photohetrotrophically on glucose and fructose.^23^

#### 2.3.7 *Plectonema*

White and Shilo^85^ discover that *Plectonema boryanum* (UTEX 594^86^) can use a variety of substrates for chemoheterotrophic growth in the dark, including ribose, sucrose, mannitol, maltose, glucose, fructose, and casamino acids. Out of all substances tested (each at a concentration of 10 mM), ribose supports the fastest growth in the dark (5 days doubling time) and also enables photoheterotrophic growth in the presence of moderate light and DCMU. The dark growth generation times on the other substances are as follows: sucrose or mannitol (6 days), maltose (10 days), glucose (12 days), and fructose (13 days). In contrast to the other substances, casamino acids alone do not cause dark growth, but in combination with ribose the doubling time is shortened by 50% from 5 days to 2.5 days.^85^ The photoheterotrophic growth of *Plectonema boryanum* (referred to in ref (23) as LPP strain PCC 73110) on glucose, fructose, ribose, and sucrose is later confirmed by Rippka et al.^23^*Plectonema notatum* (described by Kenyon et al.,^87^ also named *Plectonema boryanum* UTEX 581^86^ or *P*. *boryanum* UTEX 1542,^84^ or the LPP strain PCC 6306^23^) and *Plectonema* sp. UTEX 1541 (described by Starr,^84^ also named LPP strain PCC 6402^23^) behaves in the same way.^23^ Raboy et al.^88^ observed that glucose-dependent dark growth of *Plectonema boryanum* starts after an adaptation phase required for glucose incorporation to start. Later Raboy and Padan^89^ discovered that glucose is actively transported into the cells of this strain. Hiraide et al.^71^ identified a dark-adapted mutant strain *dg5* of *Leptolyngbya boryana* (Hiraide et al.^71^ call the strain *Plectonema boryanum*) that shows faster growth in the dark on 30 mM glucose in the BG11 medium^23^ buffered with 20 mM HEPES–KOH at pH = 7.5. The genome analysis of *dg5* reveals a mutation in the *cytM* gene that encodes Cyt *c*_M_.

#### 2.3.8 *Schizothrix*

*Schizothrix calcicola* MAN^87^ grows photoheterotrophically on glucose, fructose, sucrose, and ribose^23^ (classified as LPP strain PCC 7004).

### 2.4 Heterocyst-Forming Cyanobacteria

In this subchapter we combine the former sections IV (Nostocales forming linear filaments)^23^ and V (Stigonematales forming branching filaments),^23^ as they form one monophyletic group.^40^ Only Stigonematales, but not Nostocales, are considered to be of monophyletic origin.^90^ For phylogenetic reasons,^40^*Chroococcidiopsis* is also mentioned in this chapter.

#### 2.4.1 *Anabaena*

Sahu and Adhikary^91^ studied different organic carbon sources for an undefined *Anabaena* sp. strain isolated by Sahu et al.^92^ Fructose, but particularly lactose, enhances the growth of this strain in the light, while mannose, sodium acetate, and xylose inhibit the growth of the strain; however, none of the latter three substances completely inhibit the growth. In the dark, fructose, lactose, acetate, and mannose are stimulating, with acetate being beneficial only within the first 12 days. Lactose is the most effective substrate in both light and dark conditions, whereas xylose has the most negative effect on growth in light and no stimulating effect in the dark. Regarding nitrogen fixation in the light, acetate, mannose, and xylose all reduce the frequency of occurrence of heterocysts within a filament.^91^

*Anabaena variabilis* ATCC 29413 has been known for many years to grow in the dark at low (5 mM) fructose concentrations.^93^ It was later shown that the same sugar decreases the doubling time from 24 to 8 h, and heterocyst frequency, nitrogenase activity, and dark respiration are increased by fructose.^94^ The latter effect was reported decades ago, when Kratz and Myers^56^ found increased dark respiration with 100 mM fructose, 100 mM sucrose, and 100 mM maltose but an even stronger effect with 100 mM glucose. Schmetterer et al.^95^ reported that fructose-based dark growth is strictly dependent on an *aa*_3_-type cytochrome *c* oxidase homologous to that in mitochondria, since the deletion of the *coxBAC* operon abolishes this ability. Ungerer et al.^27^ discovered the genes responsible for fructose transport: the *frtABC* operon, which encodes the subunits of the transporter (Figure 1) and is under the control of a repressor encoded by the upstream *frtR* gene. Deletion of *frtR* abolishes dark growth on fructose, and fructose at a concentration greater than 1 mM becomes toxic to *frtR*^–^ in the light.^27^ Rapp et al.^75^ demonstrated that the FrtABC fructose transporter also imports the bioactive inhibitory molecule 7dSh into the cell since a spontaneous mutant strain resistant toward 7dSh (25 μM within the growth medium) lacks the whole *frtRABC* locus. Consequently the same mutant strain cannot use fructose for photomixotrophic or photoheterotrophic growth anymore; however, supplying a 40-fold molar concentration of fructose (1 mM) does not relieve the toxic effect of 7dSh on the wild type.^75^

The closely related strain, *Anabaena* sp. PCC 7120, has been thought to be strictly photoautotrophic for decades^23^ and lacks homologues to the *frtRABC* genes in its genome. Therefore, Ungerer et al.^27^ introduced the fructose uptake operon from *Anabaena* sp. ATCC 29413 into *Anabaena* sp. PCC 7120, thus conferring to PCC 7120 the ability to grow at low fructose concentrations (5 mM) in the dark. Rapp et al.^75^ demonstrated that the growth of a transgenic *Anabaena* sp. PCC 7120 strain containing the *frtRABC* locus is inhibited by 25 μM 7dSh. In contrast, the wild type is not affected by the same 7dSh concentration, indicating once more that the FrtABC fructose tranporter also imports 7dSh into the cells. Stebegg et al.^96^ later discovered the capacity of *Anabaena* sp. PCC 7120 wild-type strain to grow photomixotrophically, photoheterotrophically, and chemoheterotrophically at enormously high fructose concentrations (50–200 mM), without applying any genetic manipulation. Deletion of the *coxBAC1* locus, which encodes a cytochrome *c* oxidase similar to that found in the mitochondria, results in a strain unable to grow on fructose in the dark.^96^ Since similar results have been observed for *Anabaena variabilis* ATCC 29413^95^ and LAHG of *Synechocystis* sp. PCC 6803 on glucose,^70^ the question is raised whether all chemoheterotrophic growth among cyanobacteria is dependent on a functional *aa*_3_-type cytochrome *c* oxidase. Unfortunately, this has not been tested for other strains so far. The transfer of the *gtr* gene from *Synechocystis* sp. PCC 6803 on an autonomous replicating plasmid into PCC 7120 yields a strain sensitive to glucose concentrations of 5 mM or higher. Although the photoheterotrophic growth of PCC 7120 *gtr*^+^ is increased at fructose concentrations of 10 mM up to 100 mM compared to the wild type, the new transgenic strain loses the ability to grow in the dark on fructose, and 200 mM fructose is even toxic in the light.^96,97^ Unlike fructose, glucose can only support photomixotrophic growth.^96,98,99^ The five genes *glsC*, *glsP*, *glsD*, *glsQ*, and *glsR*, encoding putative components of one or more sugar transporters have been identified, and the five single knockout mutants generated result in decreased photomixotrophic, photoheterotrophic, and chemoheterotrophic growth on fructose.^100^

#### 2.4.2 *Anabaenopsis*

*Anabaenopsis circularis*(101) grows and fixes nitrogen in the dark in the presence of glucose or fructose and to a lesser extent in the presence of sucrose or maltose, while xylose, mannose, ribose, arabinose, galactose, sorbose, rhamnose, and nonsugar compounds do not affect nitrogen fixation and growth in the dark.^102^ Rippka et al.^23^ observed photoheterotrophic growth of *A*. *circularis* (CCAP 1402/1; named *Nostoc* sp. PCC 6720 in ref (23)) on glucose and fructose but not on sucrose or ribose.

#### 2.4.3 *Calothrix*

Kenyon et al.^87^ discovered the glucose-based growth of *Calothrix parietina* 1018 (also named *Calothrix parietina* UTEX 484 by Starr^86^) in both the dark and light in the presence of DCMU. Rippka et al.^23^ could not reproduce growth on glucose but reported photoheterotrophic growth on sucrose (the strain was named *Calothrix* sp. PCC 6303 by Rippka et al.^23^). Many other *Calothrix* sp. strains were identified by Rippka et al.^23^ as facultative photoheterotrophs. *Calothrix* sp. PCC 7102 (also *Calothrix desertica*(87)) grows on glucose and fructose. *Calothrix* sp. strains PCC 7103 (also *Nodularia spaerocarpa*, Koch,^103^ or UTEX 583, Starr),^86^ PCC 7111, PCC 7204, and PCC 7415 grow on glucose and fructose but also grow on sucrose, and the growth of *Calothrix* sp. strains PCC 7111 and PCC 7204 is low on fructose. *Calothrix* sp. PCC 7426 grows on ribose and sucrose and weakly on glucose and fructose. *Calothrix* sp. PCC 7116 shows growth behavior similar to that of PCC 7426, with the only exception that growth on ribose is also poor. *Calothrix* sp. PCC 7507 only grows on fructose.^23^ The strains *Calothrix* sp. PCC 7101 and PCC 7504 are discussed in the *Tolypothrix* chapter.

*Calothrix marchica* Lemm. intermedia Rao is able to utilize many carbon sources for photomixotrophic, photoheterotrophic, and chemoheterotrophic growth, including glucose, fructose, and sucrose and to a lesser extent galactose, mannitol, and sorbitol. Continuous precultivation in the presence of sucrose increases both heterotrophic growth and nitrogen content of cultures.^104^

#### 2.4.4 *Chlorogloea* (Chlorogloeopsis)

Fay and Fogg^105^ observed that *Chlorogloea fritschii*(106−108) can fix nitrogen not only in the light but also in the dark. Fay^109^ tested various organic substances to see whether they can support the growth and nitrogen fixation of *Clorogloea fritschii* in the dark. No growth is observed in the first month in the dark regardless of the substrate offered; however, after two or three months, the cultures begin to differ. Mannitol and glucose (each at a concentration of 10 mM) allow dark growth only in the presence of nitrate, while glutamine, glycine, maltose, and especially sucrose (concentration of 10 mM for each of them) induce chemoheterotrophic growth in both the presence and the absence of bound nitrogen. Sucrose, which is the most efficient substrate in the presence and absence of bound nitrogen, is tested at various concentrations (0.1–100 mM) for growth as well as nitrogen fixation in the dark, with 10 mM showing the greatest effect on both processes. Nitrate as a nitrogen source causes 4-fold dark growth at 10 mM sucrose compared to diazotrophic conditions, while addition of ammonia halves the growth. However, glucose supports growth in the dark in the absence of bound nitrogen (but still less efficiently than 10 mM sucrose) when supplied at 10-fold concentration (i.e., 100 mM), and the addition of nitrate and ammonia increases dark growth only slightly. The growth of *Chlorogloea fritschii* on acetate, pyruvate, citrate, α-ketoglutarate, succinate, fumarate, malate, glycolate, arabinose, fructose, glutamate, and aspartate fails.^109^ Rippka et al.^23^ analyzed the facultative heterotrophy of *Chlorogloea fritschii* CCAP 1411/1 (also called SAUG 1411/1 by Koch),^103^*Chlorogloeopsis fritischii* by Mitra and Pandey,^110^ or *Clorogloeopsis* sp. PCC 6912 by Rippka et al.^23^) and *Chlorogloeopsis* sp. PCC 6718. Both strains grow on glucose, fructose, ribose, and especially on sucrose.^23^

Carr^111^ discovered that offering *Chlorogloea fritschii* acetate results in an accumulation of polyhydroxyalkanoates (PHA). Monshupanee et al.^112^ observed that supplying the growth medium with acetate, pyruvate, citrate, glucose, and fructose increases the poly-3-hydroxybutyrate (PHB) production of *Chlorogloea fritschii* TISTR 8527 under photomixotrophic conditions, and PHB production is greater in the absence of nitrate than in its presence. Acetate, which has the strongest effect on PHB production in light, is also effective in the dark under chemoheterotrophic conditions, particularly in the absence of bound nitrogen and/or phosphorus. Pyruvate, citrate, glucose, and fructose are not tested in the dark. The highest PHB production occurs when cells are preincubated under photoautotrophy to increase biomass and then transferred in the dark under chemoheterotrophic conditions in the absence of bound nitrogen and/or phosphorus.^112^

#### 2.4.5 *Chroococcidiopsis*

*Chroococcidiopsis* sp. strains PCC 6712 (also described as *Chlorogloea* sp.^55^ or *Chlorogloea* sp. CCAP 1411/2), PCC 7203 (also *Myxosarcina chroococcoides* CCAP 1451/1), PCC 7431 (*Chroococcidiopsis thermalis* strain 1964/48^80^), PCC 7432 (*Chroococcidiopsisthermalis* strain 1965/21^80^), PCC 7433 (*Chroococcidiopsis thermalis* strain 1966/27^80^), PCC 7436 (*Chroococcidiopsis cubana* strain 1965/108^80^), and PCC 7439 (*Chroococcidiopsis doonensis* strain 1968/64^80^) can grow photoheterotrophically on glucose, fructose, and sucrose. Apart from PCC 6712, the other strains are assumed by Rippka et al.^23^ to be independent isolates from the same strain. *Chroococcidiopsis* sp. PCC 7434 (*C*. *cubana* strain 1965/19^80^) can only grow on fructose but not on glucose or sucrose. *Chroococcidiopsis cyanospaera* strains 1965/25 and 1965/26 (named *Dermocarpa* sp. strains PCC 7437 and PCC 7438 in ref (23)) grow on glucose and fructose.^23^

#### 2.4.6 *Cylindrospermum*

*Cylindrospermum* sp. PCC 7417 exhibits photoheterotrophic growth on fructose and sucrose.^23^

#### 2.4.7 *Fischerella*

Rippka et al.^23^ tested several *Fischerella* strains for their ability for photoheterotrophic growth. Apart from *Fischerella* sp. PCC 73103, all other strains of the genus *Fischerella* sp. identified by Rippka et al.^23^ have before been assigned to the genus *Mastigocladus*. All of these strains are capable of photoheterotrophy on glucose or fructose while differing according to their potential growth on ribose and sucrose. The *Fischerella* sp. strains PCC 7115 (also *Mastigocladus* sp. H_2_), PCC 73103 (also *Fischerella muscicola*, Koch^103^ or CCAP 1427/1 or UTEX 1301, Starr^84^), PCC 7414 (also *Mastigocladus laminosus*), and PCC 7520 (also *Mastigocladus laminosus* I-Kris-m) grow on glucose, fructose, and sucrose. Both *Fischerella* sp. strains PCC 7521 (also *Mastigocladus laminosus* Y-16-m) and PCC 7523 (also *Mastigocladus laminosus* OH-CW-m) are able to grow on glucose, fructose, sucrose, and also on ribose, but PCC 7521 only grows weakly on ribose. *Fischerella* sp. PCC 7522 (also *Mastigocladus laminosus* NZ-86-m) grows on glucose, fructose, and ribose but not on sucrose.^23^

#### 2.4.8 *Nodularia*

*Nodularia* sp. PCC 73104 grows photoheterotrophically on glucose and fructose and weakly on sucrose.^23^

#### 2.4.9 *Nostoc*

Long ago *Nostoc muscorum* was shown to exhibit slow growth and nitrogen fixation for months in the dark when glucose is supplied, and 56 mM glucose or 29 mM sucrose increases growth of *Nostoc muscorum* in the light.^113^ Lazaroff and Vishniac^114^ named this strain *Nostoc muscorum* A (as derived from Allison et al.^113^) and tested several organic molecules for dark growth. Only glucose, fructose, and sucrose act as substrates for chemoheterotrophic growth of *Nostoc muscorum* A, whereas maltose, lactose, glycerol, cellobiose, pyruvate, succinate, citrate, lactate, acetate, urea, and extracts from yeast, beef, or malt or from *Nostoc muscorum* cells fail to do so. Lazaroff and Vishniac^114^ have also checked the interrelationship of glucose and light on the growth of *Nostoc muscorum* A by measuring the dry weight after an incubation of 22 days. 56 mM glucose supports growth in total darkness; however, light intensities in the range of 1–450 ft candles further increase cell proliferation by reaching the highest dry weight at 80 ft candles. At any light intensity tested, the presence of 56 mM glucose leads to a higher cell mass compared to the same light intensity without glucose. While 56 mM glucose or 29 mM sucrose is sufficient for dark growth, higher concentrations of either sugar or addition of the other organic molecules listed above do not further increase the growth. Only an extract from light grown *Nostoc muscorum* A cells even causes a higher dark growth when added to 56 mM glucose or 29 mM sucrose. In continuous darkness, *Nostoc muscorum* changes its morphology and becomes a mass of large undifferentiated cells. Exposure to low light intensities restores the filamentous phenotype and enhances glucose- or sucrose-dependent growth. While glucose even in the light inhibits the formation of motile filaments, no such effect is observed with sucrose or fructose.^114^

Kratz and Myers^56^ detected increased respiration in the dark when glucose, fructose, sucrose, or succinate were added. Rippka et al.^23^ reported photoheterotrophic growth of *Nostoc* sp. PCC 6314 (described by Kenyon et al.;^87^ also named *Nostoc muscorum* UTEX 1545 by Starr^84^) on sucrose but not on glucose; however, growth on sucrose is sometimes absent and probably attibuted to mutations. Vaishampayan^115^ evaluated all 20 proteinogenic amino acids plus citrulline for their potential to serve as a nitrogen and/or carbon source in a heterocystous but non-nitrogen-fixing mutant strain (*het*^+^/*nif*^–^_11_) of *Nostoc muscorum*, cultured in the modified Chu 10 medium^116^ in all experiments. The modified Chu 10 medium contains 40 mg/L Ca(NO_3_)_2_, 10 mg/L K_2_HPO_4_, 25 mg/L MgSO_4_·7H_2_O, 20 mg/L Na_2_CO_3_, 25 mg/L Na_2_SiO_3_, 3 mg/L ferric citrate, and 3 mg/mL citric acid. Gerloff et al.^116^ modified the original Chu 10 medium^117^ by replacing ferric chloride with ferric citrate and citric acid and kept the K_2_HPO_4_ concentration at 10 mg/L. DCMU inhibits heterocyst formation in the absence of a suitable carbon source.^118,119^ In a positive control experiment, 3 mM glucose supports photoheterotrophic growth in the presence of DCMU.^115^ Amino acids that allow growth in a medium free of bound nitrogen are potential sources of nitrogen, while amino acids that allow the formation of heterocysts in the presence of DCMU are potential sources of carbon. Glutamine, histidine, asparagine, trypthophan, and serine are used only as a nitrogen source. Arginine, proline, and phenylalanine serve exclusively as carbon sources, while leucine, isoleucine, lysine, methionine, valine, and citrulline serve as both carbon and nitrogen sources, as growth occurs in the presence of DCMU and in the absence of bound nitrogen. Aspartic acid, threonine, and glycine have no effect in this experiment, and glutamic acid, alanine, tyrosine, and cysteine are toxic even in the presence of ammonium and in the absence of DCMU.^115^

Hoare et al.^120^ discovered that an axenic culture of *Nostoc* sp. strain MAC, which is naturally a symbiont on the coralloid roots of the cycad *Macrozamia lucida*, can use glucose, fructose, and sucrose for chemoheterotrophic growth over a pH range from 6 to 9. The growth is further enhanced by the addition of casamino acids. Harder^121^ reported that the endophyte can grow in the dark depending on glucose, galactose, sucrose, maltose, starch, insulin, and citric acid. Rippka et al.^23^ confirmed photoheterotrophic growth of *Nostoc punctiforme* (also called *Nostoc* sp. ATCC 29133 by Rippka et al.^23^) on glucose, fructose, ribose and weakly on sucrose. Summers et al.^122^ discovered both photoheterotrophic and chemoheterotrophic growth in the presence of 50 mM fructose and concomitantly added casamino acids and identified that the *zwf* gene encoding glucose-6-phosphate dehydrogenase is essential for chemoheterotrophy. Later, Ekman et al.^28^ identified the gene *glcP* and the operon *frtA1A2BC*, which encode the transporters for both sugars and show a high degree of similarity to the corresponding genes in *Synechocystis* sp. PCC 6803 and *Anabaena* sp. ATCC 29413. While wild-type growth occurs at 5 mM fructose and to a lesser extent at 5 mM glucose (in BG11 medium where nitrate is substituted by 2.5 mM ammonium chloride), replacement of most of the *frtA1A2BC* operon with C.K3,^123^ which encodes a neomycin phosphate transferease not only abolishes fructose-based chemoheterotrophic growth but also changes growth on glucose depending on the direction of insertion of C.K3 in *frtA1A2BC*. The strain CSME1A (C.K3 inserted in the opposite direction of the operon) hardly grows on glucose anymore, while the strain CSME1B (C.K3 inserted in the same direction of the operon) grows better on glucose than the wild type. In strain CSME11 both *glcP* and adjacent *oprB*, which encodes a putative carbohydrate porine, are deleted. CSME11 does not grow on glucose and growth dependent on fructose is drastically reduced.^28^ Apart from the strains described above, Rippka et al.^23^ observed photoheterotrophy in several *Nostoc* sp. strains. PCC 7107, PCC 7416, and PCC 7423 grow on fructose, while *Nostoc* sp. strains PCC 6705 and PCC 7524 grow on sucrose. The growth of strain *Nostoc* sp. PCC 6705 on glucose is variable due to mutations accumulated through culture over multiple cell cycles. Both *Nostoc* sp. strains PCC 6302 (described by Kenyon et al.,^87^ also named *Anabaena* sp. UTEX 1551 by Starr)^84^ and PCC 6310 (described by Kenyon et al.,^87^ also named *Anabaena spiroides* UTEX 1552 by Starr^84^) grow on glucose, fructose, ribose, and sucrose, but growth of the latter strain on ribose is low.^23^ Later, Schmetterer and Flores^124^ also reported growth of *Nostoc* sp. PCC 7107 (named *Nostoc* sp. ATCC 29150 in this work) on fructose in the dark while checking the fructose uptake of this strain. Several fructose concentrations (1–333 mM) are tested. The fastest growth is observed at 33 mM, while higher concentrations are less supportive.^124^

#### 2.4.10 *Scytonema*

Rippka et al.^23^ reported photoheterotrophic growth of *Scytonema* sp. PCC 7110 on glucose, fructose, and sucrose. Similar results to those obtained for *Calothrix marchica* Lemm. Var. intermedia were discovered for *Scytonema schmidlei* de Toni indicating photomixotrophic, photoheterotrophic, and chemoheterotrophic growth on glucose, fructose, and sucrose and to a lesser extent on galactose, mannitol, and sorbitol.^104^

#### 2.4.11 *Tolypothrix*

*Tolypothrix tenuis* can grow chemoheterotrophically on glucose in the dark only when ammonia is the nitrogen source and not nitrate as in most media. Ammonia and glucose enable dark growth most efficiently at a pH of 6.1. Casamino acids^125^ even support dark growth in the absence of glucose for the first 2 weeks before the culture reaches the stationary phase. Hence casamino acids can be used as both a carbon and nitrogen source; however, coaddition of glucose as a more suitable carbon source increases growth rate further. Casamino acids are most beneficial at 30 mM, while higher concentrations are less supportive of *Tolypothrix tenius* growth. In the case of glucose, on the other hand, 50 mM has the same stimulating effect as the higher concentrations. Different amino acids are tested as nitrogen sources on their own (in combination with glucose as a carbon source) for dark growth, and arginine and phenylalanine achieve about half of the growth rate compared to casamino acids when added at 28 mM, while other amino acids are less supportive. Fructose exerts an effect similar to that of glucose, while sucrose hardly elicits dark growth. Heterotrophy causes an increase of pigments like chlorophyll a, carotenoids, and phycocyanin within a week.^126^ The growth rate on glucose is further enhanced when cultures are illuminated at low light intensities (less than 500 lx), insufficient for photoautotrophic growth. Although glucose consumption from the medium is even slightly higher in the dark than in the presence of regular light (6000 lx), much more glucose is incorporated into glycogen in the light than under dark conditions.^127^ Rippka et al.^23^ reported photoheterotrophic growth of *Tolypothrix tenuis* (referred to as *Calothrix* sp. PCC 7101 in Rippka et al.^23^) on glucose, fructose, and ribose. The same phenotype was also detected for PCC 7505 by Rippka et al.,^23^ who placed this strain in the genus *Calothrix* and assumed it just to be another isolate of PCC 7101.

#### 2.4.12 *Westiellopsis*

*Westiellopsis prolifica* can fix nitrogen in light and in the dark. Fructose, lactose, sucrose, sorbose, galactose, glucose, sodium acetate, mannitol, sorbitol, and glycerol increase growth both in the dark and in the light. When incubated in the dark, the initial stimulating effect on growth is higher for all exogenous substrates mentioned, but these substances are utilized more quickly in the light. Mannose, xylose, acetic acid, propionic acid, fructose 1,6-bisphosphate, pyruvic acid, dihydroxy acetone, and succinic acid exert photogrowth inhibitory effects.^128^

### 2.5 Prochlorophyta

In addition to the classic cyanobacteria (which, because of their phycocyanins, resemble the chloroplasts of red algae), there is a special group that (like green algae and embryophytes) has chlorophyll *b* instead of phycocyanins. Therefore, they were previously thought to be the actual ancestors of chloroplasts in green algae and land plants.^129^ However, it is now assumed that classic cyanobacteria, prochlorophyta, and chloroplast of red algae, green algae, and embryophytes have developed differently from a common ancestor. This organism originally possessed both chlorophyll *b* and phycocyanines and later lost either one or both of them during evolution.^130,131^ Although these organisms do not form a monophyletic group, they are summarized as prochlorophyta.^132,133^ The genus *Prochlorococcus* is widespread in the oceans, where it (along with *Synechococcus*) is responsible for most of the marine photosynthesis.^50−52^ For a review of *Prochlorococcus* photomixotrophy see Munoz-Marin et al.^48^*Prochlorococcus* is reported to take up glucose, which increases the expression of genes involved in glucose metabolism (e.g., *zwf*, *gnd*, *dld*).^134^ It was later shown that the gene Pro1404 (although annotated as the putative melibiose/sodium symporter *melB*) encodes a glucose transporter since insertion of Pro1404 into the *asnS* gene of *Synechococcus* sp. PCC 7942 results in a strain that imports glucose into the cell. Nevertheless, the uptake rate of the new transgenic is still below the glucose uptake rate of *Synechocystis* sp. PCC 6803.^135^ Finally, a positive correlation between Pro1404 expression and glucose levels is shown.^136^ Duhamel et al.^49^ studied the photomixoautotrophic growth of undefined *Prochlorococcus* sp. strains and observed a behavior similar to that of *Synechococcus* sp. The tested *Prochlorococcus* sp. strains also assimilated glucose, leucine, ATP and especially molecules containing nitrogen and phosphorus at a higher rate in the light compared to dark conditions or when photosystem II was inhibited.

## 3 Conclusion and Future Research

From the very beginning, cyanobacteria have mainly been presented as the inventors of plant oxygenic photosynthesis, which, however, accounts for only part of their complex metabolism. While even facultatively heterotrophic cyanobacteria show the highest growth rates under photoautotrophic conditions, the various forms of heterotrophy in this kingdom are more widespread than previously thought. In recent years, more and more strains have been either intentionally identified^23,64,74,93,120,122^ or identified by chance^96^ as potential heterotrophs.

The vast majority of cyanobacteria strains is still considered to be strictly photoautotrophic^23^ with the caveat that most of these strains have not been tested on all possible substrates. Therefore, we need to examine other potential substrates (fatty acids and amino acids) that have been rather neglected in the past. For example, Rippka et al.^23^ tested only glucose, fructose, ribose, sucrose, and in some cases also glycerol for the ability to undergo heterotrophy. Although it is unlikely that strains that cannot grow photoheterotrophically have the potential for dark chemoheterotrophic growth, it is possible that some of these strains may use the substrates tested by Rippka et al.^23^ or other compounds as growth-promoting substrates for photomixotrophy. There may also be strains classified as strictly photolithoautotrophic as no suitable conditions for heterotrophy have been identified so far. In some cases, classic substrates such as fructose can stimulate growth; however, exceptionally high concentrations not existing in nature must be offered as shown in *Anabaena* sp. PCC 7120^96^ and *Synechocystis* sp. PCC 6803 *gtr*^–^,^74^ because the substrate would not enter the cell at naturally occurring external concentrations. Since no specific transporter has been identified so far, we have to assume that a transporter for a related substance (another sugar?) will import that molecule if its external concentration is high enough. Regarding the fact that photosynthesis and respiration are intimately linked in cyanobacteria,^137,138^ the question arises whether the facultative heterotrophy of some cyanobacteria strains is a relic of heterotrophic predators before the invention of oxygenic photosynthesis and was lost in other strains or whether the facultative heterotrophy was formed *de novo* as a secondary acquisition in some strains. To answer this question, knowledge about facultative (particularly photo) heterotrophy in anoxygenic phototrophs such as purple and green bacteria needs to be increased. Recently, Matheus Carnevali et al.^139^ analyzed the genome sequences of melainabacteria and sericytochromatia, which are considered to be the organisms most closely related to cyanobacteria, although they themselves do not carry out photosynthesis.^140^ According to Matheus Carnevali et al.^139^ the ancestors of cyanobacteria, melainabacteria, and seritochromatia are probably anaerobes living on fermentation and possessing various hydrogenases. Cyanobacteria therefore became aerobic after splitting off from melainabacteria and seritochromatia. Although the capacity for growth on external organic substances may have originated independently in various strains, we think that increasing the knowledge of heterotrophy among cyanobacteria will give new insights into evolutionary processes.

Cultivation under heterotrophic conditions without oxygenic photosynthesis can also promote research on critical cell parts (e.g., parts of photosystems), where otherwise erasing the gene information would be lethal. Heterotrophic cultivation can also allow growth when light must be avoided or is unavailable for long periods (e.g., transportation in space). Our picture of cyanobacteria needs to change from pure photoautotrophs to multitrophs capable of adapting to appropriate metabolic modes, depending on the current environmental conditions.

## Supporting Information Available

Table S1 that lists all cyanobacterial strains known by date to be capable of hetrotrophic growth (PDF)
