# Supplementary material for: Heterotrophy among Cyanobacteria
Source: ACS Omega. 2023 Sep 6;8(37):33098–114. doi: 10.1021/acsomega.3c02205 (PMC10515406; doi:10.1021/acsomega.3c02205)
Supplement: Supplementary file 1 — ao3c02205_si_001.pdf [file ao3c02205_si_001.pdf]

Heterotrophy among cyanobacteria

Supporting Information

Ronald Stebegg[1,*], *Georg Schmetterer*[1], *and Annette Rompel*[1,*]

[1]Universität Wien, Fakultät für Chemie, Institut für Biophysikalische Chemie, 1090 Wien, Austria.

www.bpc.univie.ac.at

*Correspondence e-mail: ronald.stebegg@univie.ac.at; and annette.rompel@univie.ac.at

Table S1: List of strains (ordered alphabetically) capable of photomixotrophic, photoheterotrophic and chemoheterotrophic growth, PM…photomixotrophic growth, PH…photoheterotrophic growth, LAHG…light activated heterotrophic growth, CH…chemoheterotrophic growth, + growth, ++ strong growth, - no growth (for PH, LAHG and CH) or no increase compared to autotrophic growth (for PM), +/- very weak growth (for PH, LAHG and CH) or very low stimulating effect (for PM), n. t. … not tested, lx…lux, ftc…foot-candle, (P)…*gtr* on a self-replicating plasmid, (C)…*gtr* integrated into the chromosome of *Synechococcus* sp. PCC 7942.

strain	substrate	medium (the numbers in ( ) without initial S refer to the main manuscript)	temperature	illumination	PM	PH	LAHG	CH	references (refers to number in main manuscript)
Anabaena sp.	fructose	Kratz and Myers medium (56) with trace elements (S1)	24 +/-2 °C	2200 lx	+ (15 mM)	n. t.	n. t.	+ (15 mM)	91
	lactose	Kratz and Myers medium (56) with trace elements (S1)	24 +/-2 °C	2200 lx	++ (15 mM)	n. t.	n. t.	++ (15 mM)	91
	acetate	Kratz and Myers medium (56) with trace elements (S1)	24 +/-2 °C	2200 lx	- (15 mM) (inhibitory)	n. t.	n. t.	+ (15 mM) (only within first 12 days)	91
	mannose	Kratz and Myers medium (56) with trace elements (S1)	24 +/-2 °C	2200 lx	- (15 mM) (inhibitory)	n. t.	n. t.	+ (15 mM)	91
	xylose	Kratz and Myers medium (56) with trace elements (S1)	24 +/-2 °C	2200 lx	- (15 mM) (very inhibitory)	n. t.	n. t.	- (15 mM)	91
Anabaena sp. PCC 7120	fructose	Allen and Arnon medium (S2)	30 °C	50 – 80 µE m^{-2} s^{-1}	- (5 mM)	n. t.	n. t.	- (5 mM)	27
	fructose	BG11 (23)	32 °C	10 µE m^{-2} s^{-1}	+ (50 - 200 mM)	+ (50 - 200 mM)	n. t.	+ (50 – 200 mM)	96
	glucose	BG11 (23)	30 °C	160 µE m^{-2} s^{-1}	+ (33 – 133 mM)	n. t.	n. t.	n. t.	98
	glucose	BG11 (23)	32 °C	10 µE m^{-2} s^{-1}	+ (50 - 200 mM)	- (10 – 200 mM)	n. t.	- (10 – 200 mM)	96
	sucrose	BG11 (23)	30 °C	60 µE m^{-2} s^{-1}	+ (2.5 mM)	- (2.5 mM)	n. t.	- (2.5 mM)	99
Anabaena sp. PCC 7120 *frtRABC*$^{+}$	fructose	Allen and Arnon medium (S2)	30 °C	50 – 80 µE m^{-2} s^{-1}	- (no effect at 1 – 5 mM, slightly inhibiting at 50 mM)	+ (5 mM)	n. t.	+ (5 – 50 mM)	27
Anabaena sp. PCC 7120 *frtABC*$^{+}$	fructose	Allen and Arnon medium (S2)	30 °C	50 – 80 µE m^{-2} s^{-1}	toxic (1 mM)	n. t.	n. t.	- (5 mM)	27

Anabaena sp. PCC 7120 *gtr*⁺	fructose	BG11 (23)	32 °C	10 µE m^{-2} s^{-1}	+ (50 – 100 mM), toxic (200 mM)	+ (10 - 100 mM), toxic (200 mM)	n. t.	- (10 – 200 mM)	96
	glucose	BG11 (23)	32 °C	10 µE m^{-2} s^{-1}	toxic (5 mM or higher)	toxic (5 mM or higher)	n.t	n. t.	96
Anabaena sp. PCC 7120 *coxBAC1*⁻	fructose	BG11 (23)	32 °C	10 µE m^{-2} s^{-1}	n. t.	n. t.	n. t.	- (50 – 200 mM)	96
Anabaena variabilis ATCC 29413	fructose	Allen and Arnon medium (S2)	30 °C	50 – 80 µE m^{-2} s^{-1}	n. t.	n. t.	n. t.	+ (5 mM)	93
	fructose	Allen and Arnon medium (S2)	30 °C	50 – 80 µE m^{-2} s^{-1}	+ (5 – 50 mM)	n. t.	n. t.	+ (5 – 50 mM)	27
Anabaena variabilis ATCC 29413 *ftrR*⁻	fructose	Allen and Arnon medium (S2)	30 °C	50 – 80 µE m^{-2} s^{-1}	toxic (5 – 200 mM)	n. t.	n. t.	- (5 mM)	27
Anabaena variabilis ATCC 29413 *coxBAC*⁻	fructose	BG11-0 (23)	30 °C	not stated	n. t.	n. t.	n. t.	- (20 mM)	95
Anabaenopsis circularis (*Nostoc* sp. PCC 6720)	glucose	nitrogen-free medium (102)	32 °C	no light used	n. t.	n. t.	n. t.	+ (5.6 – 39 mM)	102
	glucose	BG11-0 (23)	25 °C	< 500 lx	n. t.	+ (17 – 28 mM)	n. t.	n. t.	23
	fructose	nitrogen-free medium (102)	32 °C	not stated (PM), no light used (CH)	+ (17 mM)	n. t.	n. t.	+ (5.6 – 39 mM)	102
	fructose	BG11-0 (23)	25 °C	< 500 lx	n. t.	+ (17– 28 mM)	n. t.	n. t.	23
	sucrose	nitrogen-free medium (102)	32 °C	no light used	n. t.	n. t.	n. t.	+ (2.9 – 20 mM,)	102
	sucrose	BG11-0 (23)	25 °C	< 500 lx	n. t.	- (9 – 15 mM,)	n. t.	n. t.	23
	maltose	nitrogen-free medium (102)	32 °C	no light used	n. t.	n. t.	n. t.	+ (conc. not stated)	102
	ribose	BG11-0 (23)	25 °C	< 500 lx	n. t.	- (33 mM)	n. t.	n. t.	23
Arthospira platensis (also *Spirulina platensis*)	glucose	medium according to ref 82	30 °C	80 – 160 µE m^{-2} s^{-1}	+ (11 mM)	n. t.	n. t.	n. t.	81
Calothrix marchica Lemm. Var. intermedia Rao	glucose	Allen and Arnon's nitrogen-free medium (S2)	24 +/- 2 °C	2200 lx	+ (15 – 50 mM)	+ (5 – 50 mM)	n. t.	+ (15 – 50 mM)	104
	fructose	Allen and Arnon's nitrogen-free medium (S2)	24 +/- 2 °C	2200 lx	+ (5 – 50 mM)	+ (5 – 50 mM)	n. t.	+ (5 – 50 mM)	104
	sucrose	Allen and Arnon's nitrogen-free medium (S2)	24 +/- 2 °C	2200 lx	+ (7.5 – 50 mM)	+ (5 – 50 mM)	n. t.	+ (5 – 50 mM)	104
	galactose	Allen and Arnon's nitrogen-free medium (S2)	24 +/- 2 °C	2200 lx	+/- (15 mM)	+/- (15 mM)	n. t.	+/- (15 mM)	104
	mannitol	Allen and Arnon's nitrogen-free medium (S2)	24 +/- 2 °C	2200 lx	+/- (15 mM)	+/- (15 mM)	n. t.	+/- (15 mM)	104
	sorbitol	Allen and Arnon's nitrogen-free medium (S2)	24 +/- 2 °C	2200 lx	+/- (15 mM)	+/- (15 mM)	n. t.	+/- (15 mM)	104

Calothrix sp. PCC 6303	glucose	BG11 (55) at half of the normal conc. in 75 % sea water with 4 µg/l vitamin B_{12}	25 °C	100 ftc	n. t.	+ (56 mM)	n. t.	+ (56 mM)	87
	glucose	BG11-0 (23)	25 °C	< 500 lx	n. t.	- (17 – 28 mM)	n. t.	n. t.	23
	sucrose	BG11-0 (23)	25 °C	< 500 lx	n. t.	+ (9 – 15 mM)	n. t.	n. t.	23
Calothrix sp. PCC 7101 (*Tolypothrix tenuis*)	casamino acids	basal mineral medium (126)	32 °C	no light	n. t.	n. t.	n. t.	+ (3 – 55 mM)	126
	glucose	basal mineral medium (126)	32 °C	no light	n. t.	n. t.	n. t.	+ (25 – 110 mM, only if ammonia or casamino acids were used as nitrogen source)	126
	glucose	BG11-0 (23)	25 °C	< 500 lx	n. t.	+ (17 – 28 mM, 23)	n. t.	n. t.	23
	fructose	basal mineral medium (126)	32 °C	no light	n. t.	n. t.	n. t.	55 mM (ammonia or casamino acids added as nitrogen source)	126
	fructose	BG11-0 (23)	25 °C	< 500 lx	n. t.	+ (17 – 28 mM)	n. t.	n. t.	23
	ribose	BG11-0 (23)	25 °C	< 500 lx	n. t.	+ (33 mM)	n. t.	n. t.	23
	sucrose	BG11-0 (23)	25 °C	< 500 lx	n. t.	- (9 – 15 mM)	n. t.	n. t.	23
Calothrix sp. PCC 7102	glucose	BG11-0 (23)	25 °C	< 500 lx	n. t.	+ (17 – 28 mM)	n. t.	n. t.	23
	fructose	BG11-0 (23)	25 °C	< 500 lx	n. t.	+ (17 – 28 mM)	n. t.	n. t.	23
	ribose	BG11-0 (23)	25 °C	< 500 lx	n. t.	- (33 mM)	n. t.	n. t.	23
	sucrose	BG11-0 (23)	25 °C	< 500 lx	n. t.	- (9 – 15 mM)	n. t.	n. t.	23
Calothrix sp. PCC 7103	glucose	BG11-0 (23)	25 °C	< 500 lx	n. t.	+ (17 – 28 mM)	n. t.	n. t.	23
	fructose	BG11-0 (23)	25 °C	< 500 lx	n. t.	+ (17 – 28 mM)	n. t.	n. t.	23
	sucrose	BG11-0 (23)	25 °C	< 500 lx	n. t.	+ (9 – 15 mM)	n. t.	n. t.	23
	ribose	BG11-0 (23)	25 °C	< 500 lx	n. t.	- (33 mM)	n. t.	n. t.	23
Calothrix sp. PCC 7111	glucose	BG11-0 (23)	25 °C	< 500 lx	n. t.	+ (17 – 28 mM)	n. t.	n. t.	23
	fructose	BG11-0 (23)	25 °C	< 500 lx	n. t.	+/- (17 – 28 mM)	n. t.	n. t.	23
	sucrose	BG11-0 (23)	25 °C	< 500 lx	n. t.	+ (9 – 15 mM)	n. t.	n. t.	23
	ribose	BG11-0 (23)	25 °C	< 500 lx	n. t.	- (33 mM)	n. t.	n. t.	23
Calothrix sp. PCC 7116	glucose	MN (23)	25 °C	< 500 lx	n. t.	+/- (17 – 28 mM)	n. t.	n. t.	23
	fructose	MN (23)	25 °C	< 500 lx	n. t.	+/- (17 – 28 mM)	n. t.	n. t.	23
	ribose	MN (23)	25 °C	< 500 lx	n. t.	+/- (33 mM)	n. t.	n. t.	23
	sucrose	MN (23)	25 °C	< 500 lx	n. t.	+ (9 – 15 mM)	n. t.	n. t.	23
Calothrix sp. PCC 7204	glucose	BG11-0 (23)	25 °C	< 500 lx	n. t.	+ (17 – 28 mM)	n. t.	n. t.	23
	fructose	BG11-0 (23)	25 °C	< 500 lx	n. t.	+/- (17 – 28 mM)	n. t.	n. t.	23
	sucrose	BG11-0 (23)	25 °C	< 500 lx	n. t.	+ (9 – 15 mM)	n. t.	n. t.	23
	ribose	BG11-0 (23)	25 °C	< 500 lx	n. t.	- (33 mM)	n. t.	n. t.	23
Calothrix sp. PCC 7415	glucose	BG11-0 (23)	25 °C	< 500 lx	n. t.	+ (17 – 28 mM)	n. t.	n. t.	23
	fructose	BG11-0 (23)	25 °C	< 500 lx	n. t.	+ (17 – 28 mM)	n. t.	n. t.	23
	sucrose	BG11-0 (23)	25 °C	< 500 lx	n. t.	+ (9 – 15 mM)	n. t.	n. t.	23
	ribose	BG11-0 (23)	25 °C	< 500 lx	n. t.	- (33 mM)	n. t.	n. t.	23
Calothrix sp. PCC 7426	glucose	MN (23)	25 °C	< 500 lx	n. t.	+/- (17 – 28 mM)	n. t.	n. t.	23
	fructose	MN (23)	25 °C	< 500 lx	n. t.	+/- (17 – 28 mM)	n. t.	n. t.	23
	ribose	MN (23)	25 °C	< 500 lx	n. t.	+ (33 mM)	n. t.	n. t.	23

	sucrose	MN (23)	25 °C	< 500 lx	n. t.	+ (9 – 15 mM)	n. t.	n. t.	23
Calothrix sp. PCC 7504	glucose	BG11-0 (23)	25 °C	< 500 lx	n. t.	+ (17 – 28 mM)	n. t.	n. t.	23
	fructose	BG11-0 (23)	25 °C	< 500 lx	n. t.	+ (17 – 28 mM)	n. t.	n. t.	23
	ribose	BG11-0 (23)	25 °C	< 500 lx	n. t.	+ (33 mM)	n. t.	n. t.	23
	sucrose	BG11-0 (23)	25 °C	< 500 lx	n. t.	- (9 – 15 mM)	n. t.	n. t.	23
Calothrix sp. PCC 7507	fructose	BG11-0 (23)	25 °C	< 500 lx	n. t.	+ (17 – 28 mM)	n. t.	n. t.	23
	glucose	BG11-0 (23)	25 °C	< 500 lx	n. t.	- (17 – 28 mM)	n. t.	n. t.	23
	sucrose	BG11-0 (23)	25 °C	< 500 lx	n. t.	- (9 – 15 mM)	n. t.	n. t.	23
	ribose	BG11-0 (23)	25 °C	< 500 lx	n. t.	- (33 mM)	n. t.	n. t.	23
Chamaesiphon sp. PCC 7430	glucose	BG11 (23)	25 °C	< 500 lx	n. t.	+ (17 – 28 mM)	n. t.	n. t.	23
	fructose	BG11 (23)	25 °C	< 500 lx	n. t.	+ (17 – 28 mM)	n. t.	n. t.	23
	sucrose	BG11 (23)	25 °C	< 500 lx	n. t.	+ (9 – 15 mM)	n. t.	n. t.	23
Chlorogloeopsis sp. PCC 6718	glucose	BG11 (23)	25 °C	< 500 lx	n. t.	+ (17 – 28 mM)	n. t.	n. t.	23
	fructose	BG11 (23)	25 °C	< 500 lx	n. t.	+ (17 – 28 mM)	n. t.	n. t.	23
	ribose	BG11 (23)	25 °C	< 500 lx	n. t.	+ (33 mM)	n. t.	n. t.	23
	sucrose	BG11 (23)	25 °C	< 500 lx	n. t.	++ (9 – 15 mM)	n. t.	n. t.	23
Chlorogloeopsis sp. PCC 6912 (*Chlorogloea fritschii* CCAP 1411/1)	glucose	BG11-0 (23)	25 °C	< 500 lx	n. t.	+ (17 mM – 28 mM)	n. t.	n. t.	23
	fructose	BG11-0 (23)	25 °C	< 500 lx	n. t.	+ (17 – 28 mM)	n. t.	n. t.	23
	ribose	BG11-0 (23)	25 °C	< 500 lx	n. t.	+ (33 mM)	n. t.	n. t.	23
	sucrose	BG11-0 (23)	25 °C	< 500 lx	n. t.	++ (9 – 15 mM)	n. t.	n. t.	23
Chlorogloea fritschii	sucrose	Allen and Arnon medium (S2)	30 °C	no light	n. t.	n. t.	n. t.	+ (10 mM with nitrate and N_2)	109
	maltose	Allen and Arnon medium (S2)	30 °C	no light	n. t.	n. t.	n. t.	+ (10 mM with nitrate and N_2)	109
	glutamine	Allen and Arnon medium (S2)	30 °C	no light	n. t.	n. t.	n. t.	+ (10 mM with nitrate and N_2)	109
	glycine	Allen and Arnon medium (S2)	30 °C	no light	n. t.	n. t.	n. t.	+ (10 mM with nitrate and N_2)	109
	glucose	Allen and Arnon medium (S2)	30 °C	no light	n. t.	n. t.	n. t.	+ (10 – 100 mM, 10 mM only with nitrate, 100 mM with nitrate and N_2)	109
	mannitol	Allen and Arnon medium (S2)	30 °C	no light	n. t.	n. t.	n. t.	+ (10 mM only with nitrate)	109
	acetate	Allen and Arnon medium (S2)	30 °C	no light	n. t.	n. t.	n. t.	- (10 mM)	109
	pyruvate	Allen and Arnon medium (S2)	30 °C	no light	n. t.	n. t.	n. t.	- (10 mM)	109
	citrate	Allen and Arnon medium (S2)	30 °C	no light	n. t.	n. t.	n. t.	- (10 mM)	109
	α-ketoglutarate	Allen and Arnon medium (S2)	30 °C	no light	n. t.	n. t.	n. t.	- (10 mM)	109
	succinate	Allen and Arnon medium (S2)	30 °C	no light	n. t.	n. t.	n. t.	- (10 mM)	109

	fumarate	Allen and Arnon medium (S2)	30 °C	no light	n. t.	n. t.	n. t.	- (10 mM)	109
	malate	Allen and Arnon medium (S2)	30 °C	no light	n. t.	n. t.	n. t.	- (10 mM)	109
	glycolate	Allen and Arnon medium (S2)	30 °C	no light	n. t.	n. t.	n. t.	- (10 mM)	109
	arabinose	Allen and Arnon medium (S2)	30 °C	no light	n. t.	n. t.	n. t.	- (10 mM)	109
	fructose	Allen and Arnon medium (S2)	30 °C	no light	n. t.	n. t.	n. t.	- (10 mM)	109
	glutamate	Allen and Arnon medium (S2)	30 °C	no light	n. t.	n. t.	n. t.	- (10 mM)	109
	aspartate	Allen and Arnon medium (S2)	30 °C	no light	n. t.	n. t.	n. t.	- (10 mM)	109
Chroococcidiopsis sp. PCC 6712	glucose	BG11 (23)	25 °C	< 500 lx	n. t.	+ (17 – 28 mM)	n. t.	n. t.	23
	fructose	BG11 (23)	25 °C	< 500 lx	n. t.	+ (17 – 28 mM)	n. t.	n. t.	23
	sucrose	BG11 (23)	25 °C	< 500 lx	n. t.	+ (9 – 15 mM)	n. t.	n. t.	23
Chroococcidiopsis sp. PCC 7203	glucose	BG11 (23)	25 °C	< 500 lx	n. t.	+ (17 – 28 mM)	n. t.	n. t.	23
	fructose	BG11 (23)	25 °C	< 500 lx	n. t.	+ (17 – 28 mM)	n. t.	n. t.	23
	sucrose	BG11 (23)	25 °C	< 500 lx	n. t.	+ (9 – 15 mM)	n. t.	n. t.	23
Chroococcidiopsis sp. PCC 7431	glucose	BG11 (23)	25 °C	< 500 lx	n. t.	+ (17 mM – 28 mM)	n. t.	n. t.	23
	fructose	BG11 (23)	25 °C	< 500 lx	n. t.	+ (17 – 28 mM)	n. t.	n. t.	23
	sucrose	BG11 (23)	25 °C	< 500 lx	n. t.	+ (9 – 15 mM)	n. t.	n. t.	23
Chroococcidiopsis sp. PCC 7432	glucose	BG11 (23)	25 °C	< 500 lx	n. t.	+ (17 – 28 mM)	n. t.	n. t.	23
	fructose	BG11 (23)	25 °C	< 500 lx	n. t.	+ (17 – 28 mM)	n. t.	n. t.	23
	sucrose	BG11 (23)	25 °C	< 500 lx	n. t.	+ (9 – 15 mM)	n. t.	n. t.	23
Chroococcidiopsis sp. PCC 7433	glucose	BG11 (23)	25 °C	< 500 lx	n. t.	+ (17 – 28 mM)	n. t.	n. t.	23
	fructose	BG11 (23)	25 °C	< 500 lx	n. t.	+ (17 – 28 mM)	n. t.	n. t.	23
	sucrose	BG11 (23)	25 °C	< 500 lx	n. t.	+ (9 – 15 mM)	n. t.	n. t.	23
Chroococcidiopsis sp. PCC 7434	fructose	BG11 (23)	25 °C	< 500 lx	n. t.	+ (17 – 28 mM)	n. t.	n. t.	23
	glucose	BG11 (23)	25 °C	< 500 lx	n. t.	- (17 – 28 mM)	n. t.	n. t.	23
	sucrose	BG11 (23)	25 °C	< 500 lx	n. t.	- (9 – 15 mM)	n. t.	n. t.	23
Chroococcidiopsis sp. PCC 7436	glucose	BG11 (23)	25 °C	< 500 lx	n. t.	+ (17 – 28 mM)	n. t.	n. t.	23
	fructose	BG11 (23)	25 °C	< 500 lx	n. t.	+ (17 – 28 mM)	n. t.	n. t.	23
	sucrose	BG11 (23)	25 °C	< 500 lx	n. t.	+ (9 – 15 mM)	n. t.	n. t.	23
Chroococcidiopsis sp. PCC 7439	glucose	BG11 (23)	25 °C	< 500 lx	n. t.	+ (17 – 28 mM)	n. t.	n. t.	23
	fructose	BG11 (23)	25 °C	< 500 lx	n. t.	+ (17 – 28 mM)	n. t.	n. t.	23
	sucrose	BG11 (23)	25 °C	< 500 lx	n. t.	+ (9 – 15 mM)	n. t.	n. t.	23

Cyanothece sp. ATCC 51141	glycerol	ASP-2 medium (S3) modified by (53)	30 °C	80 µE m^{-2} s^{-1}	n. t.	+ (10 mM)	n. t.	n. t.	41
	glucose	ASP-2 medium (S3) modified by (53)	30 °C	80 µE m^{-2} s^{-1}	n. t.	- (not stated)	n. t.	n. t.	41
	fructose	ASP-2 medium (S3) modified by (53)	30 °C	80 µE m^{-2} s^{-1}	n. t.	- (not stated)	n. t.	n. t.	41
	sucrose	ASP-2 medium (S3) modified by (53)	30 °C	80 µE m^{-2} s^{-1}	n. t.	- (not stated)	n. t.	n. t.	41
Cyanothece sp. ATCC 51142 (*Crocosphaera subtropica* ATCC 51142)	glycerol	ASP-2 medium (S3) modified by (53)	30 °C	80 µE m^{-2} s^{-1}	n. t.	+ (10 mM)	n. t.	n. t.	41
	glycerol	ASP-2 medium (S3) modified by (53)	30 °C	50 µE m^{-2} s^{-1}	+ (54 mM)	n. t.	n. t.	n. t.	46
	glucose	ASP-2 medium (S3) modified by (53)	30 °C	80 µE m^{-2} s^{-1}	n. t.	- (not stated)	n. t.	n. t.	41
	fructose	ASP-2 medium (S3) modified by (53)	30 °C	80 µE m^{-2} s^{-1}	n. t.	- (not stated)	n. t.	n. t.	41
	sucrose	ASP-2 medium (S3) modified by (53)	30 °C	80 µE m^{-2} s^{-1}	n. t.	- (not stated)	n. t.	n. t.	41
	glucose	ASP-2 medium (S3) modified by (53)	30 °C	50 µE m^{-2} s^{-1}	+/- (26 mM)	n. t.	n. t.	n. t.	46
	sodium pyruvate	ASP-2 medium (S3) modified by (53)	30 °C	50 µE m^{-2} s^{-1}	- (9 mM), toxic (64 mM)	n. t.	n. t.	n. t.	46
Cylindrospermum sp. PCC 7417	fructose	BG11-0 (23)	25 °C	< 500 lx	n. t.	+ (17 – 28 mM)	n. t.	n. t.	23
	sucrose	BG11-0 (23)	25 °C	< 500 lx	n. t.	+ (9 – 15 mM)	n. t.	n. t.	23
Dermocarpa sp. PCC 7301	glucose	MN (23)	25 °C	< 500 lx	n. t.	+ (17 – 28 mM)	n. t.	n. t.	23
	fructose	MN (23)	25 °C	< 500 lx	n. t.	+ (17 – 28 mM)	n. t.	n. t.	23
	sucrose	MN (23)	25 °C	< 500 lx	n. t.	+ (9 – 15 mM)	n. t.	n. t.	23
Dermocarpa sp. PCC 7304	glucose	ASN-III (23)	25 °C	< 500 lx	n. t.	+ (17 – 28 mM)	n. t.	n. t.	23
	sucrose	ASN-III (23)	25 °C	< 500 lx	n. t.	+ (9 – 15 mM)	n. t.	n. t.	23
	fructose	ASN-III (23)	25 °C	< 500 lx	n. t.	- (17 – 28 mM)	n. t.	n. t.	23
Dermocarpa sp. PCC 7437	glucose	BG11 (23)	25 °C	< 500 lx	n. t.	+ (17 – 28 mM)	n. t.	n. t.	23

	sucrose	BG11 (23)	25 °C	< 500 lx	n. t.	+ (9 – 15 mM)	n. t.	n. t.	23
	fructose	BG11 (23)	25 °C	< 500 lx	n. t.	- (17 – 28 mM)	n. t.	n. t.	23
Dermocarpa sp. PCC 7438	glucose	BG11 (23)	25 °C	< 500 lx	n. t.	+ (17 – 28 mM)	n. t.	n. t.	23
	fructose	BG11 (23)	25 °C	< 500 lx	n. t.	+ (17 – 28 mM)	n. t.	n. t.	23
	sucrose	BG11 (23)	25 °C	< 500 lx	n. t.	- (9 – 15 mM)	n. t.	n. t.	23
Dermocarpella sp. PCC 7326	glucose	MN (23)	25 °C	< 500 lx	n. t.	+ (17 – 28 mM)	n. t.	n. t.	23
	fructose	MN (23)	25 °C	< 500 lx	n. t.	+ (17 – 28 mM)	n. t.	n. t.	23
	sucrose	MN (23)	25 °C	< 500 lx	n. t.	+ (9 – 15 mM)	n. t.	n. t.	23
Fischerella sp. PCC 7115	glucose	BG11-0 (23)	37 °C	< 500 lx	n. t.	+ (17 – 28 mM)	n. t.	n. t.	23
	fructose	BG11-0 (23)	37 °C	< 500 lx	n. t.	+ (17 – 28 mM)	n. t.	n. t.	23
	sucrose	BG11-0 (23)	37 °C	< 500 lx	n. t.	+ (9 – 15 mM)	n. t.	n. t.	23
	ribose	BG11-0 (23)	37 °C	< 500 lx	n. t.	- (33 mM)	n. t.	n. t.	23
Fischerella sp. PCC 73103	glucose	BG11-0 (23)	37 °C	< 500 lx	n. t.	+ (17 – 28 mM)	n. t.	n. t.	23
	fructose	BG11-0 (23)	37 °C	< 500 lx	n. t.	+ (17 – 28 mM)	n. t.	n. t.	23
	sucrose	BG11-0 (23)	37 °C	< 500 lx	n. t.	+ (9 – 15 mM)	n. t.	n. t.	23
	ribose	BG11-0 (23)	37 °C	< 500 lx	n. t.	- (33 mM)	n. t.	n. t.	23
Fischerella sp. PCC 7414	glucose	BG11-0 (23)	37 °C	< 500 lx	n. t.	+ (17 – 28 mM)	n. t.	n. t.	23
	fructose	BG11-0 (23)	37 °C	< 500 lx	n. t.	+ (17 – 28 mM)	n. t.	n. t.	23
	sucrose	BG11-0 (23)	37 °C	< 500 lx	n. t.	+ (9 – 15 mM)	n. t.	n. t.	23
	ribose	BG11-0 (23)	37 °C	< 500 lx	n. t.	- (33 mM)	n. t.	n. t.	23
Fischerella sp. PCC 7520	glucose	BG11-0 (23)	37 °C	< 500 lx	n. t.	+ (17 – 28 mM)	n. t.	n. t.	23
	fructose	BG11-0 (23)	37 °C	< 500 lx	n. t.	+ (17 – 28 mM)	n. t.	n. t.	23
	sucrose	BG11-0 (23)	37 °C	< 500 lx	n. t.	+ (9 – 15 mM)	n. t.	n. t.	23
	ribose	BG11-0 (23)	37 °C	< 500 lx	n. t.	- (33 mM)	n. t.	n. t.	23
Fischerella sp. PCC 7521	glucose	BG11 (23)	37 °C	< 500 lx	n. t.	+ (17 – 28 mM)	n. t.	n. t.	23
	fructose	BG11-0 (23)	37 °C	< 500 lx	n. t.	+ (17 – 28 mM)	n. t.	n. t.	23
	ribose	BG11-0 (23)	37 °C	< 500 lx	n. t.	+/- (33 mM)	n. t.	n. t.	23
	sucrose	BG11-0 (23)	37 °C	< 500 lx	n. t.	+ (9 – 15 mM)	n. t.	n. t.	23
Fischerella sp. PCC 7522	glucose	BG11-0 (23)	37 °C	< 500 lx	n. t.	+ (17 – 28 mM)	n. t.	n. t.	23
	fructose	BG11-0 (23)	37 °C	< 500 lx	n. t.	+ (17 – 28 mM)	n. t.	n. t.	23
	ribose	BG11-0 (23)	37 °C	< 500 lx	n. t.	+ (33 mM)	n. t.	n. t.	23
	sucrose	BG11-0 (23)	37 °C	< 500 lx	n. t.	- (9 – 15 mM)	n. t.	n. t.	23
Fischerella sp. PCC 7523	glucose	BG11-0 (23)	37 °C	< 500 lx	n. t.	+ (17 – 28 mM)	n. t.	n. t.	23
	fructose	BG11-0 (23)	37 °C	< 500 lx	n. t.	+ (17 – 28 mM)	n. t.	n. t.	23
	ribose	BG11-0 (23)	37 °C	< 500 lx	n. t.	+ (33 mM)	n. t.	n. t.	23
	sucrose	BG11-0 (23)	37 °C	< 500 lx	n. t.	+ (9 – 15 mM)	n. t.	n. t.	23
Gloeocapsa sp. PCC 7428	glucose	BG11 (23)	25 °C	< 500 lx	n. t.	+ (17 – 28 mM)	n. t.	n. t.	23

	fructose	BG11 (23)	25 °C	< 500 lx	n. t.	+ (17 – 28 mM)	n. t.	n. t.	23
	ribose	BG11 (23)	25 °C	< 500 lx	n. t.	+ (33 mM)	n. t.	n. t.	23
	sucrose	BG11 (23)	25 °C	< 500 lx	n. t.	+ (9 – 15 mM)	n. t.	n. t.	23
	glycerol	BG11 (23)	25 °C	< 500 lx	n. t.	- (11 mM)	n. t.	n. t.	23
Gloeocapsa sp. PCC 7501	glucose	BG11 (23)	25 °C	< 500 lx	n. t.	+ (17 – 28 mM)	n. t.	n. t.	23
	fructose	BG11 (23)	25 °C	< 500 lx	n. t.	- (17 – 28 mM)	n. t.	n. t.	23
	ribose	BG11 (23)	25 °C	< 500 lx	n. t.	- (33 mM)	n. t.	n. t.	23
	sucrose	BG11 (23)	25 °C	< 500 lx	n. t.	- (9 – 15 mM)	n. t.	n. t.	23
	glycerol	BG11 (23)	25 °C	< 500 lx	n. t.	- (11 mM)	n. t.	n. t.	23
LPP strain PCC 7113	sucrose	BG11 (23)	25 °C	< 500 lx	n. t.	+ (9 – 15 mM)	n. t.	n. t.	23
	glucose	BG11 (23)	25 °C	< 500 lx	n. t.	- (17 – 28 mM)	n. t.	n. t.	23
	fructose	BG11 (23)	25 °C	< 500 lx	n. t.	- (17 – 28 mM)	n. t.	n. t.	23
	ribose	BG11 (23)	25 °C	< 500 lx	n. t.	- (33 mM)	n. t.	n. t.	23
	glycerol	BG11 (23)	25 °C	< 500 lx	n. t.	- (11 mM)	n. t.	n. t.	23
LPP strain PCC 7114	glucose	BG11 (23)	25 °C	< 500 lx	n. t.	+ (17 – 28 mM)	n. t.	n. t.	23
	sucrose	BG11 (23)	25 °C	< 500 lx	n. t.	+ (9 – 15 mM)	n. t.	n. t.	23
	fructose	BG11 (23)	25 °C	< 500 lx	n. t.	- (17 – 28 mM)	n. t.	n. t.	23
	ribose	BG11 (23)	25 °C	< 500 lx	n. t.	- (33 mM)	n. t.	n. t.	23
	glycerol	BG11 (23)	25 °C	< 500 lx	n. t.	- (11 mM)	n. t.	n. t.	23
LPP strain PCC 7123	glucose	BG11 (23)	25 °C	< 500 lx	n. t.	+ (17 – 28 mM)	n. t.	n. t.	23
	fructose	BG11 (23)	25 °C	< 500 lx	n. t.	+ (17 – 28 mM)	n. t.	n. t.	23
	sucrose	BG11 (23)	25 °C	< 500 lx	n. t.	+ (9 – 15 mM)	n. t.	n. t.	23
	ribose	BG11 (23)	25 °C	< 500 lx	n. t.	- (33 mM)	n. t.	n. t.	23
	glycerol	BG11 (23)	25 °C	< 500 lx	n. t.	- (11 mM)	n. t.	n. t.	23
LPP strain PCC 7124	glucose	BG11 (23)	25 °C	< 500 lx	n. t.	+ (17 – 28 mM)	n. t.	n. t.	23
	fructose	BG11 (23)	25 °C	< 500 lx	n. t.	+ (17 – 28 mM)	n. t.	n. t.	23
	sucrose	BG11 (23)	25 °C	< 500 lx	n. t.	+ (9 – 15 mM)	n. t.	n. t.	23
	ribose	BG11 (23)	25 °C	< 500 lx	n. t.	- (33 mM)	n. t.	n. t.	23
	glycerol	BG11 (23)	25 °C	< 500 lx	n. t.	- (11 mM)	n. t.	n. t.	23
LPP strain PCC 7410	glucose	BG11 (23)	25 °C	< 500 lx	n. t.	+ (17 – 28 mM)	n. t.	n. t.	23
	fructose	BG11 (23)	25 °C	< 500 lx	n. t.	+ (17 – 28 mM)	n. t.	n. t.	23
	ribose	BG11 (23)	25 °C	< 500 lx	n. t.	+ (33 mM)	n. t.	n. t.	23
	sucrose	BG11 (23)	25 °C	< 500 lx	n. t.	+ (9 – 15 mM)	n. t.	n. t.	23
	glycerol	BG11 (23)	25 °C	< 500 lx	n. t.	- (11 mM)	n. t.	n. t.	23
LPP strain PCC 7505	glucose	BG11 (23)	25 °C	< 500 lx	n. t.	+ (17 – 28 mM)	n. t.	n. t.	23
	fructose	BG11 (23)	25 °C	< 500 lx	n. t.	+ (17 – 28 mM)	n. t.	n. t.	23
	ribose	BG11 (23)	25 °C	< 500 lx	n. t.	+ (33 mM)	n. t.	n. t.	23
	sucrose	BG11 (23)	25 °C	< 500 lx	n. t.	+ (9 – 15 mM)	n. t.	n. t.	23
	glycerol	BG11 (23)	25 °C	< 500 lx	n. t.	- (11 mM)	n. t.	n. t.	23
Lyngbya lagerheimii (strain Montauk, 39, and LPP strain PCC 7104, 23)	glucose	ASP-2 medium (S3) modified by (53)	30 °C	10 ftc/40 µW cm^{-2} (LAHG) 350 ftc (PM)	- (55 mM)	n. t.	+ (55 mM)	+/- (55 mM)	30
	glucose	BG11 (23)	25 °C	< 500 lx	n. t.	+ (17 – 28 mM)	n. t.	n. t.	23
	fructose	BG11 (23)	25 °C	< 500 lx	n. t.	+ (17 – 28 mM)	n. t.	n. t.	23

	sucrose	ASP-2 medium (S3) modified by (53)	30 °C	10 ftc/40 µW cm^{-2}	n. t.	n. t.	+/- (conc. not stated)	n. t.	30
	sucrose	BG11 (23)	25 °C	< 500 lx	n. t.	+ (9 – 15 mM)	n. t.	n. t.	23
	formate	ASP-2 medium (S3) modified by (53)	30 °C	10 ftc/40 µW cm^{-2}	n. t.	n. t.	- (11 – 22 mM)	n. t.	30
	acetate	ASP-2 medium (S3) modified by (53)	30 °C	10 ftc/40 µW cm^{-2}	n. t.	n. t.	- (8 – 17 mM)	n. t.	30
	pyruvate	ASP-2 medium (S3) modified by (53)	30 °C	10 ftc/40 µW cm^{-2}	n. t.	n. t.	- (6 – 11 mM)	n. t.	30
	succinate	ASP-2 medium (S3) modified by (53)	30 °C	10 ftc/40 µW cm^{-2}	n. t.	n. t.	- (4 – 8 mM)	n. t.	30
	ribose	BG11 (23)	25 °C	< 500 lx	n. t.	- (33 mM)	n. t.	n. t.	23
	glycerol	BG11 (23)	25 °C	< 500 lx	n. t.	- (11 mM)	n. t.	n. t.	23
Lyngbya sp. PCC 7419	glucose	MN (23)	25 °C	< 500 lx	n. t.	+/- (17 – 28 mM)	n. t.	n. t.	23
	fructose	MN (23)	25 °C	< 500 lx	n. t.	- (17 – 28 mM)	n. t.	n. t.	23
	sucrose	MN (23)	25 °C	< 500 lx	n. t.	- (9 – 15 mM)	n. t.	n. t.	23
	ribose	MN (23)	25 °C	< 500 lx	n. t.	- (33 mM)	n. t.	n. t.	23
	glycerol	MN (23)	25 °C	< 500 lx	n. t.	- (11 mM)	n. t.	n. t.	23
Microcoleus vaginatus (LPP strain PCC 7407, 23)	glucose	BG11 (23)	25 °C	< 500 lx	n. t.	+ (17 – 28 mM)	n. t.	n. t.	23
	fructose	BG11 (23)	25 °C	< 500 lx	n. t.	+ (17 – 28 mM)	n. t.	n. t.	23
	ribose	BG11 (23)	25 °C	< 500 lx	n. t.	+ (33 mM)	n. t.	n. t.	23
	sucrose	BG11 (23)	25 °C	< 500 lx	n. t.	- (9 – 15 mM)	n. t.	n. t.	23
	glycerol	BG11 (23)	25 °C	< 500 lx	n. t.	- (11 mM)	n. t.	n. t.	23
Myxosarcina sp. PCC 7312	sucrose	MN (23)	25 °C	< 500 lx	n. t.	+ (9 – 15 mM)	n. t.	n. t.	23
	glucose	MN (23)	25 °C	< 500 lx	n. t.	- (17 – 28 mM)	n. t.	n. t.	23
	fructose	MN (23)	25 °C	< 500 lx	n. t.	- (17 – 28 mM)	n. t.	n. t.	23
Myxosarcina sp. PCC 7325	glucose	MN (23)	25 °C	< 500 lx	n. t.	+ (17 – 28 mM)	n. t.	n. t.	23
	sucrose	MN (23)	25 °C	< 500 lx	n. t.	+ (9 – 15 mM)	n. t.	n. t.	23
	fructose	MN (23)	25 °C	< 500 lx	n. t.	- (17 – 28 mM)	n. t.	n. t.	23
Nodularia sp. PCC 73104	glucose	BG11-0 (23)	25 °C	< 500 lx	n. t.	+ (17 – 28 mM)	n. t.	n. t.	23
	fructose	BG11-0 (23)	25 °C	< 500 lx	n. t.	+ (17 – 28 mM)	n. t.	n. t.	23
	sucrose	BG11-0 (23)	25 °C	< 500 lx	n. t.	+/- (9 – 15 mM)	n. t.	n. t.	23
Nostoc muscorum A	glucose	medium according to ref 113	24 – 27 °C	175 – 350 ftc	+ (56 mM)	n. t.	n. t.	+ (56 mM)	113

	glucose	medium 2 (114)	24 +/- 2 °C	0 – 450 ftc	+ (56 mM)	n. t.	+ (56 mM)	+ (56 mM)	114
	sucrose	medium according to ref 113	24 – 27 °C	175 – 350 ftc	+ (29 mM)	n. t.	n. t.	n. t.	113
	sucrose	medium 2 (114)	24 +/- 2 °C	no light	n. t.	n.t.	n. t.	+ (29 mM)	114
	fructose	medium 2 (114)	24 +/- 2 °C	no light	n. t.	n. t.	n. t.	+ (not stated)	114
	maltose	medium 2 (114)	24 +/- 2 °C	no light	n. t.	n. t.	n. t.	- (not stated)	114
	lactose	medium 2 (114)	24 +/- 2 °C	no light	n. t.	n. t.	n. t.	- (not stated)	114
	glycerol	medium 2 (114)	24 +/- 2 °C	no light	n. t.	n. t.	n. t.	- (not stated)	114
	cellobiose	medium 2 (114)	24 +/- 2 °C	no light	n. t.	n. t.	n. t.	- (not stated)	114
	pyruvate	medium 2 (114)	24 +/- 2 °C	no light	n. t.	n. t.	n. t.	- (not stated)	114
	succinate	medium 2 (114)	24 +/- 2 °C	no light	n. t.	n. t.	n. t.	- (not stated)	114
	citrate	medium 2 (114)	24 +/- 2 °C	no light	n. t.	n. t.	n. t.	- (not stated)	114
	lactate	medium 2 (114)	24 +/- 2 °C	no light	n. t.	n. t.	n. t.	- (not stated)	114
	acetate	medium 2 (114)	24 +/- 2 °C	no light	n. t.	n. t.	n. t.	- (not stated)	114
	urea	medium 2 (114)	24 +/- 2 °C	no light	n. t.	n. t.	n. t.	- (not stated)	114
Nostoc muscorum	glucose	modified Chu 10 medium (116)	28 +/- 2°C	10 W m^{-2}	n. t.	+ (3 mM)	n. t.	n. t.	115
	arginine	modified Chu 10 medium (116)	28 +/- 2°C	10 W m^{-2}	n. t.	+ (50 p. p. m.)	n. t.	n. t.	115
	proline	modified Chu 10 medium (116)	28 +/- 2°C	10 W m^{-2}	n. t.	+ (50 p. p. m.)	n. t.	n. t.	115
	phenylalanine	modified Chu 10 medium (116)	28 +/- 2°C	10 W m^{-2}	n. t.	+ (50 p. p. m.)	n. t.	n. t.	115
	leucine	modified Chu 10 medium (116)	28 +/- 2°C	10 W m^{-2}	n. t.	+ (50 p. p. m.)	n. t.	n. t.	115
	isoleucine	modified Chu 10 medium (116)	28 +/- 2°C	10 W m^{-2}	n. t.	+ (50 p. p. m.)	n. t.	n. t.	115
	lysine	modified Chu 10 medium (116)	28 +/- 2°C	10 W m^{-2}	n. t.	+ (50 p. p. m.)	n. t.	n. t.	115
	methionine	modified Chu 10 medium (116)	28 +/- 2°C	10 W m^{-2}	n. t.	+ (50 p. p. m.)	n. t.	n. t.	115
	valine	modified Chu 10 medium (116)	28 +/- 2°C	10 W m^{-2}	n. t.	+ (50 p. p. m.)	n. t.	n. t.	115
	citrulline	modified Chu 10 medium (116)	28 +/- 2°C	10 W m^{-2}	n. t.	+ (50 p. p. m.)	n. t.	n. t.	115
	glutamine	modified Chu 10 medium (116)	28 +/- 2°C	10 W m^{-2}	n. t.	- (50 p. p. m.)	n. t.	n. t.	115
	histidine	modified Chu 10 medium (116)	28 +/- 2°C	10 W m^{-2}	n. t.	- (50 p. p. m.)	n. t.	n. t.	115
	asparagine	modified Chu 10 medium (116)	28 +/- 2°C	10 W m^{-2}	n. t.	- (50 p. p. m.)	n. t.	n. t.	115
	tryptophan	modified Chu 10 medium (116)	28 +/- 2°C	10 W m^{-2}	n. t.	- (50 p. p. m.)	n. t.	n. t.	115
	serine	modified Chu 10 medium (116)	28 +/- 2°C	10 W m^{-2}	n. t.	- (50 p. p. m.)	n. t.	n. t.	115

	aspartic acid	modified Chu 10 medium (116)	28 +/- 2°C	10 W m^{-2}	n. t.	- (50 p. p. m.)	n. t.	n. t.	115
	threonine	modified Chu 10 medium (116)	28 +/- 2°C	10 W m^{-2}	n. t.	- (50 p. p. m.)	n. t.	n. t.	115
	glycine	modified Chu 10 medium (116)	28 +/- 2°C	10 W m^{-2}	n. t.	- (50 p. p. m.)	n. t.	n. t.	115
	glutamic acid	modified Chu 10 medium (116)	28 +/- 2°C	10 W m^{-2}	n. t.	toxic (50 p. p. m.)	n. t.	n. t.	115
	alanine	modified Chu 10 medium (116)	28 +/- 2°C	10 W m^{-2}	n. t.	toxic (50 p. p. m.)	n. t.	n. t.	115
	tyrosine	modified Chu 10 medium (116)	28 +/- 2°C	10 W m^{-2}	n. t.	toxic (50 p. p. m.)	n. t.	n. t.	115
	cysteine	modified Chu 10 medium (116)	28 +/- 2°C	10 W m^{-2}	n. t.	toxic (50 p. p. m.)	n. t.	n. t.	115
Nostoc muscorum UTEX 1545 (also *Nostoc* sp. PCC 6314)	sucrose	BG11-0 (23)	25 °C	< 500 lx	n. t.	+/- (9 mM – 15 mM)	n. t.	n. t.	23
	glucose	BG11-0 (23)	25 °C	< 500 lx	n. t.	- (17 – 28 mM)	n. t.	n. t.	23
	fructose	BG11-0 (23)	25 °C	< 500 lx	n. t.	- (17 – 28 mM)	n. t.	n. t.	23
	ribose	BG11-0 (23)	25 °C	< 500 lx	n. t.	- (33 mM)	n. t.	n. t.	23
Nostoc punctiforme. ATCC 29133 (PCC 73102)	glucose	BG11-0 (23)	25 °C	< 500 lx	n. t.	+ (17 – 28 mM)	n. t.	n.t	23
	glucose	BG11-0 + 2.5 mM NH_4Cl + 5 mM TES-NaOH (pH 0 7.5)	30 °C	25 µE m^{-2} s^{-1}	n. t.	n. t.	n. t.	+ (5 mM)	28
	fructose	BG11-0 (23)	25 °C	< 500 lx	n. t.	+ (17 – 50 mM)	n. t.	n. t.	23
	fructose	buffered medium according to ref S4	28 °C	13 W m^{-2}	n. t.	+ (50 mM if 2.5 mM NH_4Cl and 0.5 g l^{-1} casamino acids are coadded)	n. t.	+ (50 mM if 2.5 mM NH_4Cl and 0.5 g l^{-1} casamino acids are coadded)	122
	fructose	BG11-0 + 2.5 mM NH_4Cl + 5 mM TES-NaOH (pH 0 7.5)	30 °C	25 µE m^{-2} s^{-1}	n. t.	n. t.	n. t.	+ (5 mM)	28
	ribose	BG11-0 (23)	25 °C	< 500 lx	n. t.	+ (33 mM)	n. t.	n. t.	23
	sucrose	BG11-0 (23)	25 °C	< 500 lx	n. t.	+/- (9 – 15 mM)	n. t.	n. t.	23
Nostoc punctiforme. ATCC 29133 CSME1A	glucose	BG11-0+ 2.5 mM NH_4Cl + 5 mM TES-NaOH (pH = 7.5)	30 °C	25 µE m^{-2} s^{-1}	n. t.	n. t.	n. t.	- (5 mM)	28
	fructose	BG11-0 + 2.5 mM NH_4Cl + 5 mM TES-NaOH (pH = 7.5)	30 °C	25 µE m^{-2} s^{-1}	n. t.	n. t.	n. t.	- (5 mM)	28

Nostoc punctiforme. ATCC 29133 CSME1B	glucose	BG11-0 + 2.5 mM NH_4Cl + 5 mM TES-NaOH (pH = 7.5)	30 °C	25 µE m^{-2} s^{-1}	n. t.	n. t.	n. t.	++ (5 mM)	28
	fructose	BG11-0 + 2.5 mM NH_4Cl + 5 mM TES-NaOH (pH = 7.5)	30 °C	25 µE m^{-2} s^{-1}	n. t.	n. t.	n. t.	- (5 mM)	28
Nostoc punctiforme. ATCC 29133 CSME111	glucose	BG11-0 + 2.5 mM NH_4Cl + 5 mM TES-NaOH (pH = 7.5)	30 °C	25 µE m^{-2} s^{-1}	n. t.	n. t.	n. t.	- (5 mM)	28
	fructose	BG11-0 + 2.5 mM NH_4Cl + 5 mM TES-NaOH (pH = 7.5)	30 °C	25 µE m^{-2} s^{-1}	n. t.	n. t.	n. t.	+/- (5 mM)	28
Nostoc sp. MAC	glucose	Cg 10 medium (S5)	37 °C	520 ftc (PM) 32 ftc (LAHG)	- (10 mM)	n. t.	+ (10 mM)*	+ (10 – 50 mM)* * (increased if 200 mg l^{-1} casamino acids are coadded)	120
	fructose	Cg 10 medium (S5)	37 °C	520 ftc 32 ftc (LAHG)	- (10 mM)	n. t.	+ (10 mM)	+ (10 mM)	120
	sucrose	Cg 10 medium (S5)	37 °C	520 ftc 32 ftc (LAHG)	- (10 mM)	n. t.	n. t.	+ (10 mM)	120
Nostoc sp. PCC 6302	glucose	BG11-0 (23)	25 °C	< 500 lx	n. t.	+ (17 – 28 mM)	n. t.	n. t.	23
	fructose	BG11-0 (23)	25 °C	< 500 lx	n. t.	+ (17 – 28 mM)	n. t.	n. t.	23
	ribose	BG11-0 (23)	25 °C	< 500 lx	n. t.	+ (33 mM)	n. t.	n. t.	23
	sucrose	BG11-0 (23)	25 °C	< 500 lx	n. t.	+ (9 – 15 mM)	n. t.	n. t.	23
Nostoc sp. PCC 6310	glucose	BG11-0 (23)	25 °C	< 500 lx	n. t.	+ (17 – 28 mM)	n. t.	n. t.	23
	fructose	BG11-0 (23)	25 °C	< 500 lx	n. t.	+ (17 – 28 mM)	n. t.	n. t.	23
	ribose	BG11-0 (23)	25 °C	< 500 lx	n. t.	+/- (33 mM)	n. t.	n. t.	23
	sucrose	BG11-0 (23)	25 °C	< 500 lx	n. t.	+ (9 – 15 mM)	n. t.	n. t.	23
Nostoc sp. PCC 6705	sucrose	BG11-0 (23)	25 °C	< 500 lx	n. t.	+/- (9 – 15 mM)	n. t.	n. t.	23
	glucose	BG11-0 (23)	25 °C	< 500 lx	n. t.	- (17 – 28 mM)	n. t.	n. t.	23
	fructose	BG11-0 (23)	25 °C	< 500 lx	n. t.	- (17 – 28 mM)	n. t.	n. t.	23
	ribose	BG11-0 (23)	25 °C	< 500 lx	n. t.	- (33 mM)	n. t.	n. t.	23
Nostoc sp. PCC 7107 (also *Nostoc* sp. ATCC 29150)	fructose	BG11-0 (23)	25 °C	< 500 lx	n. t.	+ (17 – 28 mM)	n. t.	n. t.	23
	fructose	$^{1}/_{8}$ Allen and Arnon's medium (S2) + 2.5 mM $NaNO_3$ + 2.5 mM KNO_3	no light	30 mM	n. t.	n. t.	n. t.	+ (1 - 333 mM)	124
	glucose	BG11-0 (23)	25 °C	< 500 lx	n. t.	- (17 – 28 mM)	n. t.	n. t.	23
	ribose	BG11-0 (23)	25 °C	< 500 lx	n. t.	- (33 mM)	n. t.	n. t.	23
	sucrose	BG11-0 (23)	25 °C	< 500 lx	n. t.	- (9 – 15 mM)	n. t.	n. t.	23

Nostoc sp. PCC 7416	fructose	BG11-0 (23)	25 °C	< 500 lx	n. t.	+ (17 – 28 mM)	n. t.	n. t.	23
	glucose	BG11-0 (23)	25 °C	< 500 lx	n. t.	- (17 – 28 mM)	n. t.	n. t.	23
	ribose	BG11-0 (23)	25 °C	< 500 lx	n. t.	- (33 mM)	n. t.	n. t.	23
	sucrose	BG11-0 (23)	25 °C	< 500 lx	n. t.	- (9 – 15 mM)	n. t.	n. t.	23
Nostoc sp. PCC 7423	fructose	BG11-0 (23)	25 °C	< 500 lx	n. t.	+ (17 – 28 mM)	n. t.	n. t.	23
	glucose	BG11-0 (23)	25 °C	< 500 lx	n. t.	- (17 – 28 mM)	n. t.	n. t.	23
	ribose	BG11-0 (23)	25 °C	< 500 lx	n. t.	- (33 mM)	n. t.	n. t.	23
	sucrose	BG11-0 (23)	25 °C	< 500 lx	n. t.	- (9 – 15 mM)	n. t.	n. t.	23
Nostoc sp. PCC 7524	sucrose	BG11-0 (23)	25 °C	< 500 lx	n. t.	+ (9 – 15 mM)	n. t.	n. t.	23
	glucose	BG11-0 (23)	25 °C	< 500 lx	n. t.	- (17 – 28 mM)	n. t.	n. t.	23
	fructose	BG11-0 (23)	25 °C	< 500 lx	n. t.	- (17 – 28 mM)	n. t.	n. t.	23
	ribose	BG11-0 (23)	25 °C	< 500 lx	n. t.	- (33 mM)	n. t.	n. t.	23
Oscillatoria williamsii MEV (LPP strain PCC 7105, 23)	glucose	MN (23)	25 °C	< 500 lx	n. t.	+/- (17 – 28 mM)	n. t.	n. t.	23
	fructose	MN (23)	25 °C	< 500 lx	n. t.	+/- (17 – 28 mM)	n. t.	n. t.	23
	sucrose	MN (23)	25 °C	< 500 lx	n. t.	+ (9 – 15 mM)	n. t.	n. t.	23
	glycerol	MN (23)	25 °C	< 500 lx	n. t.	+ (11 mM)	n. t.	n. t.	23
	ribose	MN (23)	25 °C	< 500 lx	n. t.	- (33 mM)	n. t.	n. t.	23
Oscillatoria sp. PCC 6407	glucose	BG11 (23)	25 °C	< 500 lx	n. t.	+/- (17 – 28 mM)	n. t.	n. t.	23
	fructose	BG11-0 (23)	25 °C	< 500 lx	n. t.	- (17 – 28 mM)	n. t.	n. t.	23
Oscillatoria sp. PCC 6412	glucose	BG11 (23)	25 °C	< 500 lx	n. t.	+/- (17 – 28 mM)	n. t.	n. t.	23
	fructose	BG11 (23)	25 °C	< 500 lx	n. t.	+/- (17 – 28 mM)	n. t.	n. t.	23
Oscillatoria sp. PCC 6506	glucose	BG11 (23)	25 °C	< 500 lx	n. t.	+/- (17 – 28 mM)	n. t.	n. t.	23
	fructose	BG11-0 (23)	25 °C	< 500 lx	n. t.	- (17 – 28 mM)	n. t.	n. t.	23
Oscillatoria sp. PCC 6602	glucose	BG11 (23)	25 °C	< 500 lx	n. t.	+ (17 – 28 mM)	n. t.	n. t.	23
	fructose	BG11 (23)	25 °C	< 500 lx	n. t.	+ (17 – 28 mM)	n. t.	n. t.	23
Oscillatoria sp. PCC 7515	glucose	BG11 (23)	25 °C	< 500 lx	n. t.	+ (17 – 28 mM)	n. t.	n. t.	23
	fructose	BG11 (23)	25 °C	< 500 lx	n. t.	+ (17 – 28 mM)	n. t.	n. t.	23
Phormidium sp. UTEX 1540 (PCC 6409, 23)	glucose	BG11 (23)	25 °C	< 500 lx	n. t.	+ (17 – 28 mM)	n. t.	n. t.	23
	fructose	BG11 (23)	25 °C	< 500 lx	n. t.	+ (17 – 28 mM)	n. t.	n. t.	23
	ribose	BG11 (23)	25 °C	< 500 lx	n. t.	- (33 mM)	n. t.	n. t.	23
	sucrose	BG11 (23)	25 °C	< 500 lx	n. t.	- (9 – 15 mM)	n. t.	n. t.	23
	glycerol	BG11 (23)	25 °C	< 500 lx	n. t.	- (11 mM)	n. t.	n. t.	23
Plectonema boryanum UTEX 581 (LPP strain 6306, 23)	glucose	BG11 (23)	25 °C	< 500 lx	n. t.	+ (17 – 28 mM)	n. t.	n. t.	23
	fructose	BG11 (23)	25 °C	< 500 lx	n. t.	+ (17 – 28 mM)	n. t.	n. t.	23
	ribose	BG11 (23)	25 °C	< 500 lx	n. t.	+ (33 mM)	n. t.	n. t.	23
	sucrose	BG11 (23)	25 °C	< 500 lx	n. t.	+ (9 – 15 mM)	n. t.	n. t.	23
	glycerol	BG11 (23)	25 °C	< 500 lx	n. t.	- (11 mM)	n. t.	n. t.	23

Plectonema boryanum UTEX 594 (*Leptolyngbya boryana* or LPP strain PCC 73110, 23)	ribose	modified Chu No 10 medium (S6)	33 °C	1300 lx (PH), 85 lx (LAHG)	n. t.	+ (10 mM)	+ (10 mM)	+ (10 mM)	85
	ribose	BG11 (23)	25 °C	< 500 lx	n. t.	+ (33 mM)	n. t.	n. t.	23
	sucrose	modified Chu No 10 medium (S6)	33 °C	no light	n. t.	n. t.	n. t.	+ (10 mM)	85
	sucrose	BG11 (23)	25 °C	< 500 lx	n. t.	+ (9 – 15 mM)	n. t.	n. t.	23
	mannitol	modified Chu No 10 medium (S6)	33 °C	no light	n. t.	n. t.	n. t.	+ (10 mM)	85
	maltose	modified Chu No 10 medium (S6)	33 °C	no light	n. t.	n. t.	n. t.	+ (10 mM)	85
	glucose	modified Chu No 10 medium (S6);	33 °C	no light	n. t.	n. t.	n. t.	+ (10 mM)	85
	glucose	BG11 (23)	25 °C	< 500 lx	n. t.	+ (17 – 28 mM)	n. t.	n. t.	23
	fructose	modified Chu No 10 medium (S6)	33 °C	no light	n. t.	n. t.	n. t.	+ (10 mM)	85
	fructose	BG11 (23)	25 °C	< 500 lx	n. t.	+ (17 – 28 mM)	n. t.	n. t.	23
	casamino acids (only with ribose)	modified Chu No 10 medium (S6)	33 °C	85 lx	n. t.	n. t.	+ (1 g/l)	+ (1 g/l)	85
	glycerol	BG11 (23)	25 °C	< 500 lx	n. t.	- (11 mM)	n. t.	n. t.	23
Plectonema boryanum UTEX 594 *dg5* (in this paper *Leptolyngbya boryanua dg5*)	glucose	BG11 buffered with 20 mM HEPES -KOH pH = 8.2	30 °C	50 µE m^{-2} s^{-1}	+/- (30 mM)	n. t.	+ (30 mM)	+ (30 mM)	71
Plectonema sp. UTEX 1541 (LPP strain PCC 6402, 23)	glucose	BG11 (23)	25 °C	< 500 lx	n. t.	+ (17 – 28 mM)	n. t.	n. t.	23
	fructose	BG11 (23)	25 °C	< 500 lx	n. t.	+ (17 – 28 mM)	n. t.	n. t.	23
	ribose	BG11 (23)	25 °C	< 500 lx	n. t.	+ (33 mM)	n. t.	n. t.	23
	sucrose	BG11 (23)	25 °C	< 500 lx	n. t.	+ (9 – 15 mM)	n. t.	n. t.	23
	glycerol	BG11 (23)	25 °C	< 500 lx	n. t.	- (11 mM)	n. t.	n. t.	23
Pleurocapsa sp. PCC 7310	sucrose	ASN-III (23)	25 °C	< 500 lx	n. t.	+ (9 – 15 mM)	n. t.	n. t.	23
	glucose	ASN-III (23)	25 °C	< 500 lx	n. t.	- (17 – 28 mM)	n. t.	n. t.	23
	fructose	ASN-III (23)	25 °C	< 500 lx	n. t.	- (17 – 28 mM)	n. t.	n. t.	23
Pleurocapsa sp. PCC 7314	fructose	MN (23)	25 °C	< 500 lx	n. t.	+ (17 – 28 mM)	n. t.	n. t.	23
	sucrose	MN (23)	25 °C	< 500 lx	n. t.	+ (9 – 15 mM)	n. t.	n. t.	23
	glucose	MN (23)	25 °C	< 500 lx	n. t.	- (17 – 28 mM)	n. t.	n. t.	23

Pleurocapsa sp. PCC 7317	glucose	MN (23)	25 °C	< 500 lx	n. t.	+ (17 – 28 mM)	n. t.	n. t.	23
	fructose	MN (23)	25 °C	< 500 lx	n. t.	- (17 – 28 mM)	n. t.	n. t.	23
	sucrose	MN (23)	25 °C	< 500 lx	n. t.	- (9 – 15 mM)	n. t.	n. t.	23
Pleurocapsa sp. PCC 7319	fructose	MN (23)	25 °C	< 500 lx	n. t.	+ (17 – 28 mM)	n. t.	n. t.	23
	sucrose	MN (23)	25 °C	< 500 lx	n. t.	+ (9 – 15 mM)	n. t.	n. t.	23
	glucose	MN (23)	25 °C	< 500 lx	n. t.	- (17 – 28 mM)	n. t.	n. t.	23
Pleurocapsa sp. PCC 7320	glucose	MN (23)	25 °C	< 500 lx	n. t.	+ (17 – 28 mM)	n. t.	n. t.	23
	fructose	MN (23)	25 °C	< 500 lx	n. t.	+ (17 – 28 mM)	n. t.	n. t.	23
	sucrose	MN (23)	25 °C	< 500 lx	n. t.	+ (9 – 15 mM)	n. t.	n. t.	23
Pleurocapsa sp. PCC 7321	sucrose	BG11 (23)	25 °C	< 500 lx	n. t.	+ (9 – 15 mM)	n. t.	n. t.	23
	glucose	BG11 (23)	25 °C	< 500 lx	n. t.	- (17 – 28 mM)	n. t.	n. t.	23
	fructose	BG11 (23)	25 °C	< 500 lx	n. t.	- (17 – 28 mM)	n. t.	n. t.	23
Pleurocapsa sp. PCC 7322	glucose	MN (23)	25 °C	< 500 lx	n. t.	+ (17 – 28 mM)	n. t.	n. t.	23
	fructose	MN (23)	25 °C	< 500 lx	n. t.	+ (17 – 28 mM)	n. t.	n. t.	23
	sucrose	MN (23)	25 °C	< 500 lx	n. t.	+ (9 – 15 mM)	n. t.	n. t.	23
Pleurocapsa sp. PCC 7327	sucrose	BG11 (23)	37 °C	< 500 lx	n. t.	+ (9 – 15 mM)	n. t.	n. t.	23
	glucose	BG11 (23)	37 °C	< 500 lx	n. t.	- (17 – 28 mM)	n. t.	n. t.	23
	fructose	BG11 (23)	37 °C	< 500 lx	n. t.	- (17 – 28 mM)	n. t.	n. t.	23
Pleurocapsa sp. PCC 7506	glucose	MN (23)	25 °C	< 500 lx	n. t.	+ (17 – 28 mM)	n. t.	n. t.	23
	fructose	MN (23)	25 °C	< 500 lx	n. t.	- (17 – 28 mM)	n. t.	n. t.	23
	sucrose	MN (23)	25 °C	< 500 lx	n. t.	- (9 – 15 mM)	n. t.	n. t.	23
Pleurocapsa sp. PCC 7516	sucrose	MN (23)	25 °C	< 500 lx	n. t.	+ (9 – 15 mM)	n. t.	n. t.	23
	glucose	MN (23)	25 °C	< 500 lx	n. t.	- (17 – 28 mM)	n. t.	n. t.	23
	fructose	MN (23)	25 °C	< 500 lx	n. t.	- (17 – 28 mM)	n. t.	n. t.	23
Scytonema schmidlei De Toni	glucose	Allen and Arnon's nitrogen-free medium (S2)	24 +/- 2 °C	2200 lx	+ (5 – 50 mM)	+ (5 – 50 mM)	n. t.	+ (5 – 50 mM)	104
	fructose	Allen and Arnon's nitrogen-free medium (S2)	24 +/- 2 °C	2200 lx	+ (5 – 50 mM)	+ (5 – 50 mM)	n. t.	+ (5 – 50 mM)	104
	sucrose	Allen and Arnon's nitrogen-free medium (S2)	24 +/- 2 °C	2200 lx	+ (5 – 50 mM)	+ (5 – 50 mM)	n. t.	+ (5 – 50 mM)	104
	galactose	Allen and Arnon's nitrogen-free medium (S2)	24 +/- 2 °C	2200 lx	+/- (5 – 50 mM)	+/- (5 – 50 mM)	n. t.	+/- (5 – 50 mM)	104
	mannitol	Allen and Arnon's nitrogen-free medium (S2)	24 +/- 2 °C	2200 lx	+/- (5 – 50 mM)	+/- (5 – 50 mM)	n. t.	+/- (5 – 50 mM)	104

	sorbitol	Allen and Arnon's nitrogen-free medium (S2)	24 +/- 2 °C	2200 lx	+/- (5 – 50 mM)	+/- (5 – 50 mM)	n. t.	+/- (5 – 50 mM)	104
Scytonema sp. PCC 7110	glucose	BG11-0 (23)	25 °C	< 500 lx	n. t.	+ (17 – 28 mM)	n. t.	n. t.	23
	fructose	BG11-0 (23)	25 °C	< 500 lx	n. t.	+ (17 – 28 mM)	n. t.	n. t.	23
	sucrose	BG11-0 (23)	25 °C	< 500 lx	n. t.	+ (9 – 15 mM)	n. t.	n. t.	23
Schizothrix calcicola MAN<br>(LPP strain PCC 7004)	glucose	BG11 (23)	25 °C	< 500 lx	n. t.	+ (17 – 28 mM)	n. t.	n. t.	23
	fructose	BG11 (23)	25 °C	< 500 lx	n. t.	+ (17 – 28 mM)	n. t.	n. t.	23
	ribose	BG11 (23)	25 °C	< 500 lx	n. t.	+ (33 mM)	n. t.	n. t.	23
	sucrose	BG11 (23)	25 °C	< 500 lx	n. t.	+ (9 – 15 mM)	n. t.	n. t.	23
	glycerol	BG11 (23)	25 °C	< 500 lx	n. t.	- (11 mM)	n. t.	n. t.	23
Synechococcus sp. PCC 7002 (formerly called *Agmenellum quadruplicatum* PR-6)	glucose	ASP-2 medium (S3) modified by (53)	39 °C	10 ftc/40 µW cm^{-2} (LAHG)<br>350 ftc (PM)	- (44 – 55 mM)	n. t.	+/- (11 mM)<br>+ (44 – 55 mM)	- (44 – 55 mM)	30
	glucose	BG11 (23)	25 °C	< 500 lx	n. t.	+/- (17 – 28 mM)	n. t.	n. t.	23
	glucose	medium A (S7)	38 °C	not stated	n. t.	- (10 – 50 mM)	n. t.	n. t.	54
	acetate	ASP-2 medium (S3) modified by (53)	39 °C	10 ftc/40 µW cm^{-2} (LAHG)	n. t.	n. t.	- (12 mM)	n. t.	30
	glycerol	BG11 (23)	25 °C	< 500 lx	n. t.	+ (11 mM)	n. t.	n. t.	23
	glycerol	medium A (S7)	38 °C	not stated	+/- (3 – 30 mM)	+ (1 – 100 mM)	n. t.	n. t.	54
	fructose	BG11 (23)	25 °C	< 500 lx	n. t.	- (17 – 28 mM)	n. t.	n. t.	23
	fructose	medium A (S7)	38 °C	not stated	n. t.	- (10 – 50 mM)	n. t.	n. t.	54
	sucrose	BG11 (23)	25 °C	< 500 lx	n. t.	- (9 – 15 mM)	n. t.	n. t.	23
	sodium malate	medium A (S7)	38 °C	not stated	n. t.	- (10 – 50 mM)	n. t.	n. t.	54
	sodium citrate	medium A (S7)	38 °C	not stated	n. t.	- (10 – 50 mM)	n. t.	n. t.	54
Synechococcus sp. PCC 7002 G2 (formerly called *Agmenellum quadruplicatum* PR-6G2)	glycerol	not stated	38 ° C	not stated	- (3 – 30 mM)	+ (1 – 100 mM)	n. t.	n. t.	54
Synechococcus sp. PCC 7003	glucose	MN (23)	25 °C	< 500 lx	n. t.	+/- (17 – 28 mM)	n. t.	n. t.	23
	glycerol	MN (23)	25 °C	< 500 lx	n. t.	+ (11 mM)	n. t.	n. t.	23
	fructose	MN (23)	25 °C	< 500 lx	n. t.	- (17 – 28 mM)	n. t.	n. t.	23
	sucrose	MN (23)	25 °C	< 500 lx	n. t.	- (9 – 15 mM)	n. t.	n. t.	23
Synechococcus sp. PCC 7335	fructose	BG11 (23)	25 °C	< 500 lx	n. t.	+ (17 – 28 mM)	n. t.	n. t.	23
	glucose	BG11 (23)	25 °C	< 500 lx	n. t.	- (17 – 28 mM)	n. t.	n. t.	23
	sucrose	BG11 (23)	25 °C	< 500 lx	n. t.	- (9 – 15 mM)	n. t.	n. t.	23
	glycerol	BG11 (23)	25 °C	< 500 lx	n. t.	- (11 mM)	n. t.	n. t.	23

Synechococcus sp. PCC 73109	glucose	BG11 (23)	25 °C	< 500 lx	n. t.	+/- (17 – 28 mM)	n. t.	n. t.	23
	fructose	BG11 (23)	25 °C	< 500 lx	n. t.	+/- (17 – 28 mM)	n. t.	n. t.	23
	glycerol	BG11 (23)	25 °C	< 500 lx	n. t.	+ (11 mM)	n. t.	n. t.	23
	sucrose	BG11 (23)	25 °C	< 500 lx	n. t.	- (9 – 15 mM)	n. t.	n. t.	23
Synechococcus sp. PCC 7511	glucose	BG11 (23)	25 °C	< 500 lx	n. t.	+ (17 – 28 mM)	n. t.	n. t.	23
	sucrose	BG11 (23)	25 °C	< 500 lx	n. t.	+ (9 – 15 mM)	n. t.	n. t.	23
	fructose	BG11 (23)	25 °C	< 500 lx	n. t.	- (17 – 28 mM)	n. t.	n. t.	23
	glycerol	BG11 (23)	25 °C	< 500 lx	n. t.	- (11 mM)	n. t.	n. t.	23
Synechococcus sp. PCC 7942	glucose	BG11 (23)	30 °C	diurnal light (25 µE $m^{-2}s^{-1}$)	+ (28 mM)	n. t.	n. t.	n. t.	60
	sucrose	BG11 (23)	30 °C	diurnal light (25 µE $m^{-2}s^{-1}$)	+ (15 mM)	n. t.	n. t.	n. t.	60
	xylose	BG11 (23)	30 °C	diurnal light (25 µE $m^{-2}s^{-1}$)	+ (28 mM)	n. t.	n. t.	n. t.	60
Synechococcus sp. PCC 7942 *galP*$^+$	glucose	BG11 (23)	30 °C	diurnal light (25 µE $m^{-2}s^{-1}$)	++ (28 mM)	n. t.	n. t.	n. t.	60
Synechococcus sp. PCC 7942 *Glut1*$^+$	glucose	BG11 (23)	30 °C	diurnal light (25 µE $m^{-2}s^{-1}$)	- (28 mM)	n. t.	n. t.	n. t.	60
Synechococcus sp. PCC 7942 *gtr*$^+$ (P)	glucose	modified Allen's medium (S8)	32 °C	50 – 60 µE m^{-2} s^{-1} (3000 lx)	n. t.	+ (56 mM), plasmid could not be maintained stably	n. t.	n. t.	39
Synechococcus sp. PCC 7942 *gtr*$^+$ (C)	glucose	modified Allen's medium (S8)	32 °C	50 – 60 µE m^{-2} s^{-1} (3000 lx)	toxic (56 mM)	toxic (56 mM)	n. t.	n. t.	39
	glucose	BG11 (23)	30 °C	diurnal light (25 µE $m^{-2}s^{-1}$)	+ (28 mM) (until 12 h), toxic (after 12 h)	n. t.	n. t.	n. t.	60
Synechococcus sp. PCC 7942 *cscKB*$^+$	sucrose	BG11 (23)	30 °C	diurnal light (25 µE $m^{-2}s^{-1}$)	++ (15 mM)	n. t.	n. t.	n. t.	60
Synechococcus sp. PCC 7942 *xylE*$^+$	xylose	BG11 (23)	30 °C	diurnal light (25 µE $m^{-2}s^{-1}$)	- (28 mM)	n. t.	n. t.	n. t.	60
Synechococcus sp. PCC 7942 *xylEAB*$^+$	xylose	BG11 (23)	30 °C	diurnal light (25 µE $m^{-2}s^{-1}$)	++ (28 mM)	n. t.	n. t.	n. t.	60
Synechococcus sp. PCC 7942 *glf*$^+$	glucose	BG11 (23)	30 °C	no light	n. t.	n. t.	n. t.	+ (500 µM)	58
	fructose	BG11 (23)	30 °C	no light	n. t.	n. t.	n. t.	+ (500 µM)	58
Synechocystis sp. PCC 6702	glucose	BG11 (23)	25 °C	< 500 lx	n. t.	+ (17 – 28 mM)	n. t.	n. t.	23
	sucrose	BG11 (23)	25 °C	< 500 lx	n. t.	- (9 – 15 mM)	n. t.	n. t.	23
	glycerol	BG11 (23)	25 °C	< 500 lx	n. t.	- (11 mM)	n. t.	n. t.	23
Synechocystis sp. PCC 6714	glucose	BG11 (23)	25 °C	< 500 lx	n. t.	+ (17 – 28 mM)	n.t	n. t.	23
	glucose	Allen's medium with 2x nitrate conc. (S9)	34 °C	70 µE m^{-2} s^{-1} (3500 lx)	n. t.	+ (56 mM)	n. t.	+ (56 mM)	64
	sucrose	BG11 (23)	25 °C	< 500 lx	n. t.	- (9 – 15 mM)	n. t.	n. t.	23
	glycerol	BG11 (23)	25 °C	< 500 lx	n. t.	- (11 mM)	n. t.	n. t.	23

Synechocystis sp. PCC 6803	glucose	BG11 (23)	25 °C	< 500 lx	n. t.	+ (17 – 28 mM)	n. t.	n. t.	23
	glucose	BG11 (23)	30 °C	not stated	-/slightly toxic (10 mM) (a glucose tolerant variant has been isolated by (69))	+ (10 mM)	n. t.	n. t.	68
(tolerant variant of *Synechocystis* sp. PCC 6803 (69))	glucose	BG11 (23) + 5 mM TES pH = 8.0	31 +/- 2 °C	40 µmol m^{-2} s^{-1}	n. t.	n. t.	+ (5 mM)	- (5 mM)	29
(tolerant variant of *Synechocystis* sp. PCC 6803 (69))	glucose	BG11 (23) + 6 mM HEPES according to ref 65	31 +/- 2 °C	40 µmol m^{-2} s^{-1}	n. t.	n. t.	+ (56 mM)	+/- (56 mM) (only for the first 6 days, then declining)	29
	sucrose	BG11 (23)	25 °C	< 500 lx	n. t.	- (9 – 15 mM)	n. t.	n. t.	23
	glycerol	BG11 (23)	25 °C	< 500 lx	n. t.	- (11 mM)	n. t.	n. t.	23
	fructose	BG11 (23)	30 °C	not stated	toxic (10 mM)	n. t.	n. t.	n. t.	68
	xylose	BG11 (23)	30 °C	50 µE m^{-2} s^{-1} for 1 h per day (LAHG)	n. t.	n. t.	- (5 – 50 mM)	n. t.	63
Synechocystis sp. PCC 6803 *gtr*$^-$	glucose	BG11 (23)	30 °C	not stated	not toxic (10 mM)	- (10 mM)	n. t.	n. t.	68
	fructose	BG11 (23)	30 °C	not stated	not toxic (10 mM)	n. t.	n. t.	n. t.	68
	fructose	BG11 (23)	32 °C	10 µE m^{-2} s^{-1}	+ (50 – 200 mM)	+ (50 – 200 mM)	-	-	74
Synechocystis sp. PCC 6803 *psbA*$^-$ (of glucose tolerant variant of *Synechocystis* sp. PCC 6803 (69))	glucose	BG11 (23) + 5 mM TES pH = 8.0	31 +/- 2 °C	40 µmol m^{-2} s^{-1}	n. t.	n. t.	+ (5 mM)	n. t.	29
Synechocystis sp. PCC 6803 *coxBAC*$^-$	glucose	BG11 (23)	32 °C	40 µE m^{-2} s^{-1}	n. t.	+ (10 mM)	- (10 mM)	n. t.	70
Synechocystis sp. PCC 6803 *cytM*$^-$	glucose	BG11 (23) buffered with 20 mM HEPES-KOH pH = 8.2	30 °C	50 µE m^{-2} s^{-1}	+ (5 mM)	n. t.	+ (5 mM)	+ (5 mM)	71
Synechocystis sp. PCC 6803 *xylAB*$^+$	glucose	BG11 (23)	30 °C	50 µE m^{-2} s^{-1} for 1h per day (LAHG)	n. t.	n. t.	+ (5.6 mM)	n. t.	63
	xylose	BG11 (23)	30 °C	50 µE m^{-2} s^{-1} for 1h per day (LAHG)	n. t.	n. t.	- (5 – 50 mM)	n. t.	63
Synechocystis sp. PCC 6805	glucose	BG11 (23)	25 °C	< 500 lx	n. t.	+ (17 – 28 mM)	n. t.	n. t.	23
	sucrose	BG11 (23)	25 °C	< 500 lx	n. t.	- (9 – 15 mM)	n. t.	n. t.	23
	glycerol	BG11 (23)	25 °C	< 500 lx	n. t.	- (11 mM)	n. t.	n. t.	23
Synechocystis sp. PCC 6806	glucose	BG11 (23)	25 °C	< 500 lx	n. t.	+ (17 – 28 mM)	n. t.	n. t.	23
	sucrose	BG11 (23)	25 °C	< 500 lx	n. t.	- (9 – 15 mM)	n. t.	n. t.	23
	glycerol	BG11 (23)	25 °C	< 500 lx	n. t.	- (11 mM)	n. t.	n. t.	23

Synechocystis sp. PCC 6905	glucose	BG11 (23)	25 °C	< 500 lx	n. t.	+ (17 – 28 mM)	n. t.	n. t.	23
	sucrose	BG11 (23)	25 °C	< 500 lx	n. t.	- (9 – 15 mM)	n. t.	n. t.	23
	glycerol	BG11 (23)	25 °C	< 500 lx	n. t.	- (11 mM)	n. t.	n. t.	23
Synechocystis sp. PCC 6906	glucose	BG11 (23)	25 °C	< 500 lx	n. t.	+/- (17 – 28 mM)	n. t.	n. t.	23
	glycerol	BG11 (23)	25 °C	< 500 lx	n. t.	+ (11 mM)	n. t.	n. t.	23
	sucrose	BG11 (23)	25 °C	< 500 lx	n. t.	- (9 – 15 mM)	n. t.	n. t.	23
Synechocystis sp. PCC 7201	glucose	BG11 (23)	25 °C	< 500 lx	n. t.	+ (17 – 28 mM)	n. t.	n. t.	23
	sucrose	BG11 (23)	25 °C	< 500 lx	n. t.	- (9 – 15 mM)	n. t.	n. t.	23
	glycerol	BG11 (23)	25 °C	< 500 lx	n. t.	- (11 mM)	n. t.	n. t.	23
Synechocystis sp. PCC 7509	glucose	BG11 (23)	25 °C	< 500 lx	n. t.	+ (17 – 28 mM)	n. t.	n. t.	23
	sucrose	BG11 (23)	25 °C	< 500 lx	n. t.	+ (9 – 15 mM)	n. t.	n. t.	23
	glycerol	BG11 (23)	25 °C	< 500 lx	n. t.	- (11 mM)	n. t.	n. t.	23
Thermosynechococcus elongatus	glucose	BG11 (23)	48 °C	20 µE m^{-2} s^{-1}	+ (50 mM)	+ (50 mM)	n. t.	n. t.	79
	fructose	BG11 (23)	48 °C	20 µE m^{-2} s^{-1}	+ (50 mM)	+ (50 mM)	+ (50 mM), 15 min 25 µE m^{-2} s^{-1} illumination	+ (50 mM)	79
	galactose	BG11 (23)	48 °C	20 µE m^{-2} s^{-1}	+ (50 mM)	+ (50 mM)	n. t.	n. t.	79
	lactose	BG11 (23)	48 °C	20 µE m^{-2} s^{-1}	+ (35 mM)	+ (35 mM)	n. t.	n. t.	79
	sucrose	BG11 (23)	48 °C	20 µE m^{-2} s^{-1}	+ (35 mM)	+ (35 mM)	n. t.	n. t.	79
	arabinose	BG11 (23)	48 °C	20 µE m^{-2} s^{-1}	- (75 mM)	- (75 mM)	n. t.	n. t.	79
	maltose	BG11 (23)	48 °C	20 µE m^{-2} s^{-1}	- (35 mM)	- (35 mM)	n. t.	n. t.	79
	trehalose	BG11 (23)	48 °C	20 µE m^{-2} s^{-1}	- (35 mM)	- (35 mM)	n. t.	n. t.	79
	xylose	BG11 (23)	48 °C	20 µE m^{-2} s^{-1}	inhibitory (75 mM)	- (75 mM)	n. t.	n. t.	79
Westiellopsis prolifica	fructose	Allen and Arnon medium (S2)	28 +/- 2°C	2200 lx	++ (11 mM)	n. t.	n. t.	++ (11 mM)	128
	lactose	Allen and Arnon's medium (S2)	28 +/- 2°C	2200 lx	++ (5.8 mM)	n. t.	n. t.	++ (5.8 mM)	128
	sucrose	Allen and Arnon's medium (S2)	28 +/- 2°C	2200 lx	++ (5.5 mM)	n. t.	n. t.	++ (5.8 mM)	128
	sorbose	Allen and Arnon's medium (S2)	28 +/- 2°C	2200 lx	+ (11 mM)	n. t.	n. t.	++ (11 mM)	128
	galactose	Allen and Arnon's medium (S2)	28 +/- 2°C	2200 lx	+ (11 mM) (only for the first 15 days)	n. t.	n. t.	++ (11 mM)	128
	glucose	Allen and Arnon's medium (S2)	28 +/- 2°C	2200 lx	+ (11 mM)	n. t.	n. t.	++ (11 mM)	128
	sodium acetate	Allen and Arnon's medium (S2)	28 +/- 2°C	2200 lx	+ (24 mM) (only for the first 10 days)	n. t.	n. t.	++ (24 mM)	128

	mannitol	Allen and Arnon's medium (S2)	28 +/- 2°C	2200 lx	+ (11 mM)	n. t.	n. t.	++ (11 mM)	128
	sorbitol	Allen and Arnon's medium (S2)	28 +/- 2°C	2200 lx	+ (11 mM)	n. t.	n. t.	++ (11 mM)	128
	glycerol	Allen and Arnon's medium (S2)	28 +/- 2°C	2200 lx	+ (22 mM) (only for the first 10 days)	n. t.	n. t.	+ (22 mM)	128
	mannose	Allen and Arnon's medium (S2)	28 +/- 2°C	2200 lx	inhibitory (11 mM)	n. t.	n. t.	+ (11 mM)	128
	xylose	Allen and Arnon's medium (S2)	28 +/- 2°C	2200 lx	inhibitory (11 mM)	n. t.	n. t.	+ (11 mM)	128
	acetic acid	Allen and Arnon's medium (S2)	28 +/- 2°C	2200 lx	inhibitory (33 mM)	n. t.	n. t.	- (33 mM)	128
	propionic acid	Allen and Arnon's medium (S2)	28 +/- 2°C	2200 lx	inhibitory (27 mM)	n. t.	n. t.	- (27 mM)	128
	fructose 1,6-bisphosphate	Allen and Arnon's medium (S2)	28 +/- 2°C	2200 lx	inhibitory (6 mM)	n. t.	n. t.	+ (6 mM)	128
	pyruvic acid	Allen and Arnon's medium (S2)	28 +/- 2°C	2200 lx	Inhibitory (23 mM)	n. t.	n. t.	- (23 mM)	128
	dihydroxy acetone	Allen and Arnon's medium (S2)	28 +/- 2°C	2200 lx	Inhibitory (22 mM)	n. t.	n. t.	+ (22 mM)	128
	succinic acid	Allen and Arnon's medium (S2)	28 +/- 2°C	2200 lx	Inhibitory (17 mM)	n. t.	n. t.	+ (17 mM)	128
Xenococcus sp. PCC 7307	glucose	MN (23)	25 °C	< 500 lx	n. t.	+ (17 – 28 mM)	n. t.	n. t.	23
	sucrose	MN (23)	25 °C	< 500 lx	n. t.	+ (9 – 15 mM)	n. t.	n. t.	23
	fructose	MN (23)	25 °C	< 500 lx	n. t.	- (17 – 28 mM)	n. t.	n. t.	23

References

S1. Fogg, G. E. Growth and heterocyst production in *Anabaena cylindrica* Lemm. II. In relation to carbon and nitrogen metabolism. *Ann. Bot.* **1949**, *13*, 241-259, DOI: 10.1038/220810b0

S2. Allen, M. B.; Arnon, D. I. Studies on nitrogen-fixing blue-green algae. I. Growth and nitrogen fixation by *Anabaena cylindrica* Lemm. *Plant Physiol.* **1955**, *30*, 366-372, DOI: 10.1104/pp.30.4.366

S3. Provasoli, L.; McLaughlin, J. J.; Droop, M. R. The development of artificial media for marine algae. *Arch. Mikrobiol.* **1957**, *25*, 392-428. DOI: 10.1007/BF00446694

S4. Cohen, M. F.; Wallis, J. G.; Campbell, E. L.; Meeks, J. C. Transposon mutagenesis of *Nostoc* sp. strain ATCC 29133, a filamentous cyanobacterium with multiple cellular differentiation alternatives. *Microbiology* (Reading) **1994**, *140*, 3233-3240, DOI: 10.1099/13500872-140-12-3233

S5. Van Baalen, C. Further observations on growth of single cells of coccoid blue-green algae. *J. Phycol.* **1967**, *3*, 154-157, DOI: 10.1111/j.1529-8817.1967.tb04650.x

S6. Safferman, R. S.; Morris, M. E. Observations on the occurrence, distribution, and seasonal incidence of blue-green algal viruses. *Appl. Microbiol.* 1967, *15*, 1210-1222, DOI: 10.1128/am.15.5.1219-1222.1967

S7. Stevens, S. E. Jr.; Patterson, C. O. P.; Myers, J. The production of hydrogen peroxide by blue-green algae: a survey. *J. Phycol.* **1973**, *9*, 427-430, DOI: 10.1128/jb.165.2.654-656.1986

S8. Panoff, J. M.; Priem; B., Morvan, H.; Joset, F. Sulphated exopolysaccharides produced by two unicellular strains of cyanobacteria, *Synechocystis* PCC 6803 and 6714. *Arch. Microbiol.* **1988**, *150*, 558-563, DOI: 10.1007/BF00408249

S9. Joset-Espardellier, F.; Astier, C.; Evan, E. H.; Carr, N. G. Cyanobacteria grown under photoautotrophic, photoheterotrophic and heterotrophic regimes: sugar metabolism and carbon dioxide fixation. *FEMS Microbiol. Lett.* **1978**, *4*, 261-264, DOI: 10.1111/j.1574-6968.1978.tb02875.x
